# Personalized Network‐Guided Neuromodulation Enhances Human Working Memory

**DOI:** 10.1002/advs.202523009

**Published:** 2026-06-12

**Authors:** Ahsan Khan, Hongming Li, Camille Blaine, Julie Grier, Ethan Hammett, Almaris Figueroa‐Gonzalez, Sarai Garcia, Romain Duprat, Justin Reber, Joseph Deluisi, Christos Davatzikos, Theodore D. Satterthwaite, Yong Fan, Desmond J. Oathes

**Affiliations:** ^1^ Center For Brain Imaging and Stimulation Department of Psychiatry Perelman School of Medicine University of Pennsylvania Philadelphia Pennsylvania USA; ^2^ Center For Neuromodulation in Depression and Stress Department of Psychiatry Perelman School of Medicine University of Pennsylvania Philadelphia Pennsylvania USA; ^3^ Department of Education and Psychology Academy of Wellness and Human Development Faculty of Arts and Social Sciences Hong Kong Baptist University Kowloon Tong Hong Kong SAR China; ^4^ Center For Biomedical Image Computing and Analytics Department of Radiology Perelman School of Medicine University of Pennsylvania Philadelphia Pennsylvania USA; ^5^ Center for AI and Data Science for Integrated Diagnostics University of Pennsylvania Philadelphia PA USA; ^6^ Lifespan Informatics & Neuroimaging Center Department of Psychiatry Perelman School of Medicine University of Pennsylvania Philadelphia Pennsylvania USA; ^7^ Lifespan Brain Institute Children's Hospital of Philadelphia University of Pennsylvania Philadelphia Pennsylvania USA; ^8^ Penn Brain Science Translation Innovation and Modulation Center University of Pennsylvania Philadelphia Pennsylvania USA; ^9^ Departments of Neuroscience Bioengineering Neurology and Neurosurgery Perelman School of Medicine University of Pennsylvania Philadelphia Pennsylvania USA

**Keywords:** brain state decoding, functional brain networks, personalized neuromodulation, transcranial magnetic stimulation, working memory

## Abstract

The next frontier in cognitive neuromodulation is defined by personalized and adaptive protocols, necessitating approaches tailored to individual functional neuroanatomy and brain‐state fluctuations. Here, we introduce an adaptive neuromodulation framework that integrates individualized network targeting with real‐time decoding of brain states to precisely target working memory functional networks. Using concurrent transcranial magnetic stimulation (TMS) and functional magnetic resonance imaging (fMRI), we first mapped participant‐specific networks and identified personalized targets. A real‐time decoder then tracked stimulation‐evoked neural dynamics to empirically determine the optimal frequency (i.e., the best‐performing within a tested set of 5, 10, and 20 Hz) and a corresponding suboptimal frequency for each individual. In a multi‐session crossover study, only the optimal‐frequency stimulation significantly improved working memory, with the decoder's output predicting behavioral gains. A key finding is the substantial inter‐individual variability in the optimal frequency, providing evidence against the notion of a universal “best” frequency. Our results demonstrate that cognitive enhancement is governed by the precise interaction between stimulation target and frequency. This work provides a causal demonstration of personalized, network‐based neuromodulation and offers proof of concept for a generalizable, biomarker‐driven framework, representing a step toward advancing cognitive therapeutics.

**Trial Registration**: This study is registered at ClinicalTrials.gov (identifier: NCT04402294).

## Graphical abstract

A personalized neuromodulation framework combining individualized functional brain network targeting with real‐time neural decoding is introduced. Using concurrent TMS–fMRI, participant‐specific stimulation targets and optimal frequencies are identified. Only optimal‐frequency stimulation improves working memory across sessions. Results demonstrate that both target and frequency must be individually tailored for effective cognitive enhancement.

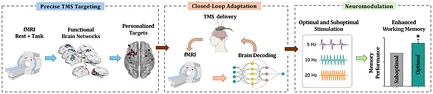

## 1 Introduction

Imagine a therapy that could sharpen your cognitive abilities or slow down cognitive decline, but it only worked for a lucky few. For the last two decades, that has been the frustrating promise of non‐invasive brain stimulation [1]. We know tools like Transcranial Magnetic Stimulation (TMS) can influence brain circuits, but their effects on crucial cognitive functions like working memory, the ability to hold and manipulate information in your mind, have been notoriously unpredictable. The problem is that we have been missing a crucial piece of the puzzle: individuality. Our brain's wiring is a unique, dynamic landscape. The very networks that power working memory, such as the fronto‐parietal and default mode networks, vary significantly from person to person and fluctuate over time [2, 3, 4]. Yet, standard methods have used a one‐size‐fits‐all approach, applying the same stimulation to the same scalp location for everyone [5, 6]. In this study, we moved beyond this static paradigm by interacting with the brain during stimulation and adjusting stimulation parameters. We first mapped each individual's unique functional architecture to identify a personalized stimulation target. Then, we used a real‐time decoder to track neural responses to stimulation, enabling the selection of the optimal frequency from the tested set for each participant. The identified frequencies were then evaluated across multiple neuromodulation sessions. We demonstrate that this personalized approach, which respects the biological individuality of each brain, produces robust and reliable improvements in working memory, providing a causal link between precisely‐tuned, network‐specific activity and cognitive enhancement.

The brain functions as a network of interconnected cortical and subcortical regions collaborating within a hierarchical structure [7, 8, 9, 10, 11, 12, 13, 14, 15]. The effect of stimulation on a target cortical region thus propagates throughout the brain, resulting in network‐level modulation [16, 17]. Accurately determining the cortical stimulation site is therefore crucial for inducing these distributed changes. Various methods have traditionally been used, including the ‘5 cm rule’ and ‘Beam F3’ method, to loosely approximate the prefrontal cortex [18]; however, these methods do not account for individual differences in brain structure and function. In contrast, functional connectivity networks (FCNs) derived from functional magnetic resonance imaging (fMRI) offer a reproducible framework for identifying individualized stimulation targets [19]. Traditionally, these networks are identified using group‐averaged FCNs derived from resting‐state fMRI data. However, FCNs can vary markedly in their spatial topography across brain states and individuals [20, 21, 22, 23, 24, 25, 26]. Recent studies indicate that individualized, sparse, non‐negative personalized functional networks (PFNs) provide improved characterization of the functional brain at the individual level [27, 28]. Additionally, task‐based fMRI enhances both within‐subject and between‐subject estimates of stable brain network representations compared to using resting state fMRI only [4]. Considering these factors, our study utilized both resting and task‐evoked brain imaging data to define PFNs as brain stimulation targets.

Besides TMS targeting, repetitive TMS (rTMS) frequency has clear neurobiological consequences [29]. Without directly measuring brain response to rTMS in humans, there remains substantial unexplained variability and even a sizable number of participants who exhibit ‘excitatory’ reactions to putative inhibitory stimulation and vice versa [30], suggesting that common rTMS excitatory frequencies vary in their cortical effects across individuals. Currently, no systematic solution is available to effectively tune TMS frequency for optimal brain stimulation of an individual brain. We expect that tuning TMS frequency based on fMRI real‐time feedback could help to personalize and therefore optimize stimulation effects on the brain and behavior. Here, we utilized brain decoding models with long short‐term (LSTM) recurrent neural networks (RNN) [31, 32] to obtain real‐time brain state readouts and to guide stimulation frequencies optimization at the individual subject level. Specifically, we extracted functional profiles from working memory task fMRI data based on PFNs, which served as features to build brain decoding models. LSTM RNNs were adopted to learn decoding mappings between the functional profiles and brain states. This approach builds on our previous work, where we demonstrated that brain decoding models built on PFNs using LSTM RNNs can accurately decode working memory and motor tasks in real time [32].

Overall, the study aimed to enhance working memory by leveraging each individual's unique brain network organization. Using PFNs to guide stimulation targets and adjusting stimulation frequencies based on real‐time brain activity, we examined whether this individualized approach could improve working memory performance across multiple sessions. We predicted that stimulation at optimal frequencies, defined by better decoder predictions, would selectively enhance working memory performance, and that the decoder's predictions would improve post‐neuromodulation training, reflecting the consolidation of network‐level changes. By combining network‐informed targeting with real‐time brain decoding, this study provides a framework for understanding how dynamic brain networks can be modulated with brain stimulation to improve cognitive performance.

## 2 Results

### 2.1 Overview of Study Design

**FIGURE 1:**
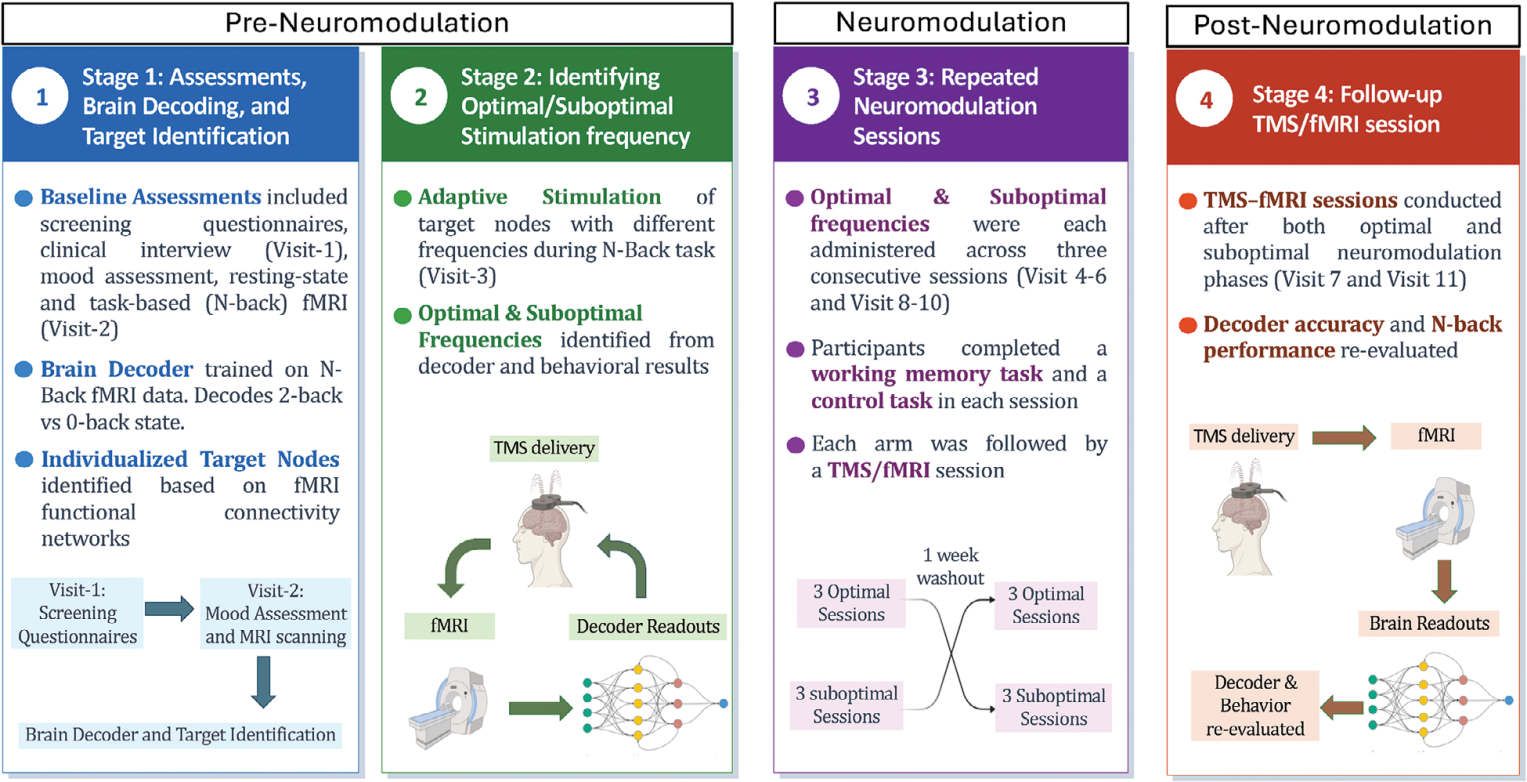
Overview of Study Design: The study comprised four sequential stages. Stage 1 involved baseline screening questionnaires and a clinical interview (Visit 1), followed by mood assessment and resting‐state and task‐based (N‐back) fMRI (Visit 2). A brain decoder was then trained on the N‐back fMRI data to classify 2‐back versus 0‐back brain states, and individualised stimulation target nodes were identified based on PFNs identified using both resting and task (N‐back) based fMRI data. Stage 2 involved adaptive rTMS stimulation of target nodes at multiple frequencies interleaved with N‐back task blocks during a TMS/fMRI session (Visit 3). Optimal and suboptimal stimulation frequencies were identified for each participant based on decoder output reflecting working memory network engagement and behavioral performance. Stage 3 employed a randomized crossover design in which optimal and suboptimal frequencies were each administered across three consecutive daily sessions (Visits 4–6 and Visits 8–10, respectively), separated by a minimum one‐week washout period. During each session, participants completed a working memory task and a processing speed control task following rTMS delivery. Stage 4 consisted of TMS/fMRI sessions conducted after each neuromodulation phase (Visits 7 and 11), during which decoder accuracy and N‐back task performance were re‐evaluated to assess the effects of neuromodulation training on working memory network engagement.

To investigate whether individually‐tailored rTMS frequencies can selectively modulate working memory network engagement and behavioral performance, we implemented an adaptive TMS‐fMRI paradigm in which stimulation frequencies were personalized for each participant prior to a randomized crossover neuromodulation intervention. The overall study design is shown in Figure 1. During the pre‐neuromodulation phase, which spanned three separate visits, participants first completed screening questionnaires and a clinical interview at Visit 1. At Visit 2, additional mood assessments were conducted, followed by structural and functional MRI scans acquired during an N‐back working memory task. Subsequently, a brain decoder was trained using the collected fMRI data, and individualized stimulation targets were identified based on PFNs during an approximately two‐week interval. Following this, participants underwent a Pre‐neuromodulation TMS/fMRI session on Visit 3 during which rTMS at different frequencies was interleaved with N‐back blocks. During this session, stimulation frequencies were classified for each individual as optimal or suboptimal based on decoder classified working memory network engagement and behavioral performance. Neuromodulation began the following day, with participants completing six sessions in a crossover design, receiving three consecutive optimal and three consecutive suboptimal neuromodulation sessions in a randomized order across two separate weeks. The optimal and suboptimal sessions were conducted with a minimum one‐week washout period between them. During each session, participants performed both a working memory task and a control task following rTMS stimulation. The working memory task used during the neuromodulation session was a delayed matching‐to‐sample (DMTS) task, in which participants were instructed to retain a visual stimulus in memory over a delay period and were subsequently tested on it. This task differed from the N‐back working memory task used during the TMS/fMRI session to evaluate whether the effects of stimulation generalize across different working memory tasks. In addition, the control task assessed processing speed and was included to test that any changes in working memory were specifically attributable to the influence of stimulation on working memory processes. Post‐Neuromodulation TMS/fMRI sessions (Visit 7 and Visit 11) assessed the effects of stimulation and network engagement after neuromodulation training.

### 2.2 Mapping Individualized Stimulation Targets

**FIGURE 2:**
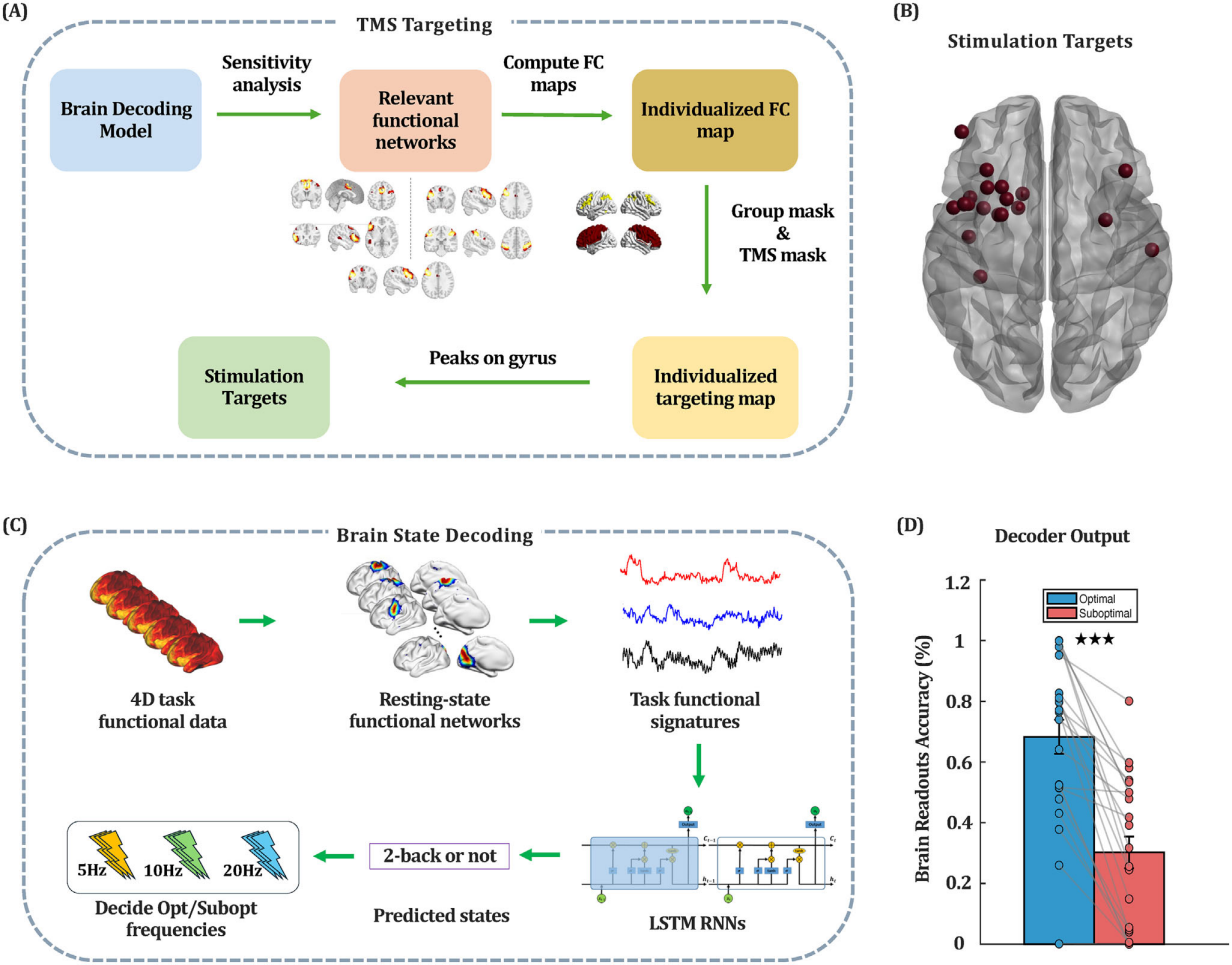
Individualized TMS Targeting and Brain Decoding Pipeline. (A) Schematic of the individualized TMS targeting approach is shown. The brain decoding model was first used to identify functional networks relevant to working memory. Subject‐specific functional connectivity (FC) maps were then derived from these networks using a combination of resting‐state and task‐based fMRI data, generating an individualised targeting map for each participant. Individualized Stimulation targets were then selected from these maps to maximise engagement of working memory‐relevant circuitry. (B) Stimulation targets identified for all participants (n = 19) overlaid on a cortical surface rendering in Montreal Neurological Institute (MNI) space, illustrating the distribution and inter‐individual variability of the individually‐derived target sites across the cohort. (C) Schematic of the brain state decoding approach, where the decoder distinguishes whether the participant is performing the 2‐back or 0‐back task, enabling selection of optimal and suboptimal stimulation frequencies. (D) Average decoder outputs during the TMS/fMRI session on Visit 3 for optimal and suboptimal stimulation frequencies. Bars represent the group means, and error bars represent ± 1 SE across participants (n = 19). Statistical comparison was performed with a two‐tailed paired‐samples t‐test: t(18) = 6.80, *p* < 0.001, Cohen's d = 1.56. ^***^ *p* < 0.001.

During the baseline measures, participants underwent MRI scanning to identify individualized TMS targets. We identified the stimulation target as the cortical brain region showing the strongest functional connectivity with working memory‐related PFNs. Specifically, we conducted a principal component analysis (PCA) on the baseline brain decoder (details provided in the methods) to determine the five PFNs most relevant to working memory task decoding. These were then used to generate individualized FC maps for target identification. We then performed functional connectivity analysis on these selected PFNs to generate an individualized targeting map. The complete pipeline for target identification is illustrated in Figure 2A, while the primary targets of all participants are shown in Figure 2B, visualized using BrainNet viewer [33]. In our study, we identified two targets for each participant to ensure that if the primary target was inaccessible for any reason with the TMS coil, the secondary target could be used as a backup (see Figure S2 for both primary and secondary targets).

### 2.3 Determining Optimal and Suboptimal Frequencies

Following the identification of individualized stimulation targets, optimal and suboptimal frequencies for stimulation were determined during a pre‐neuromodulation TMS/fMRI session. This session comprised two sequential runs of the N‐back task, Each incorporating both a 2‐back condition (the current item matches the one shown two trials earlier) and a 0‐back condition (the current item matches a specific target). During the first run, called the random loop, three putative excitatory rTMS frequencies (5, 10, and 20 Hz) were administered in a randomized order. As stimulation was delivered, the decoder estimated in real time the degree to which each frequency engaged the working memory network, operationalized as the probability that the participant was performing the 2‐back task. Higher decoder output for a specific frequency indicated stronger network engagement and was defined as the optimal frequency. It is important to note that optimal frequency refers to the stimulation frequency that produced the highest decoder‐derived network engagement or improved behavioral performance, within the predefined set of tested frequencies, and does not imply identification of a global physiological optimum. Correspondingly, suboptimal frequency refers to the frequency that produced the weakest network engagement within that set. A schematic of the full brain‐state decoding process is shown in Figure 2C. Building on these initial estimates, the second run, the informed loop, administered the frequencies in a specified order to systematically test and confirm how the identified candidate frequencies modulated network engagement. Full details of both runs are provided in Table S1.

Importantly, frequency selection was performed under real‐time constraints during the TMS–fMRI session, requiring stimulation decisions to be made within a limited time window while participants remained in the scanner. The decoder served as the primary decision tool for classifying stimulation frequencies as optimal or suboptimal. However, in 9 out of 19 participants (47%), the decoder did not produce a reliable discriminative output in real time after the random loop, and therefore could not serve as the sole basis for frequency selection. To ensure that a stimulation decision could always be made within the session, a predefined hierarchical fallback procedure was implemented whereby behavioral performance guided frequency selection. Specifically, the frequency associated with the higher hit rate was designated as optimal, with reaction time serving as a secondary tie‐breaker when hit rates were equivalent.

For subsequent analyses, decoder output was averaged across trials for each frequency to yield stable estimates of network engagement. During the informed loop, the decoder output was significantly higher under optimal than suboptimal conditions (t(18) = 6.80, p < 0.001, Cohen's d = 1.56; Figure 2D), providing strong post‐hoc validation of the frequency selection procedure. To explore potential patterns underlying decoder failure, we evaluated the temporal contrast‐to‐noise ratio (tCNR) of the WM‐relevant functional networks. Participants in whom the decoder reliably classified stimulation frequencies (Decoder group) showed higher tCNR than those in the Behavioral fallback group, a difference that approached significance (0.237 ± 0.116 vs. 0.172 ± 0.058, p = 0.075); further details are provided in Note S1 and Figure S3.

Given that the decoder was fine‐tuned in successive stages as participant data accumulated, we evaluated its discriminative performance between optimal and suboptimal stimulation at Visit 3 (pre‐neuromodulation) for each decoder version, to determine whether the findings were sensitive to the order in which fine‐tuning was performed. The difference in decoder output between optimal and suboptimal conditions, was consistently larger for optimal than suboptimal stimulation across all three versions: v1 (n = 4, Diff: optimal = 0.699 ± 0.238, suboptimal = 0.266 ± 0.149, p = 0.062, Cohen's d = 1.45), v2 (n = 5, Diff: optimal = 0.637 ± 0.052, suboptimal = 0.295 ± 0.114, p = 0.048, Cohen's d = 1.26), and v3 (n = 10, Diff: optimal = 0.680 ± 0.085, suboptimal = 0.280 ± 0.090, *p* < 0.001, Cohen's d = 1.78). Overall, these analyses demonstrate that the decoder reliably distinguished between optimal and suboptimal stimulation across successive iterations, with consistent effects across versions and increasing statistical robustness in the final model.

### 2.4 Task Performance and Decoder–Behavior Associations

**FIGURE 3:**
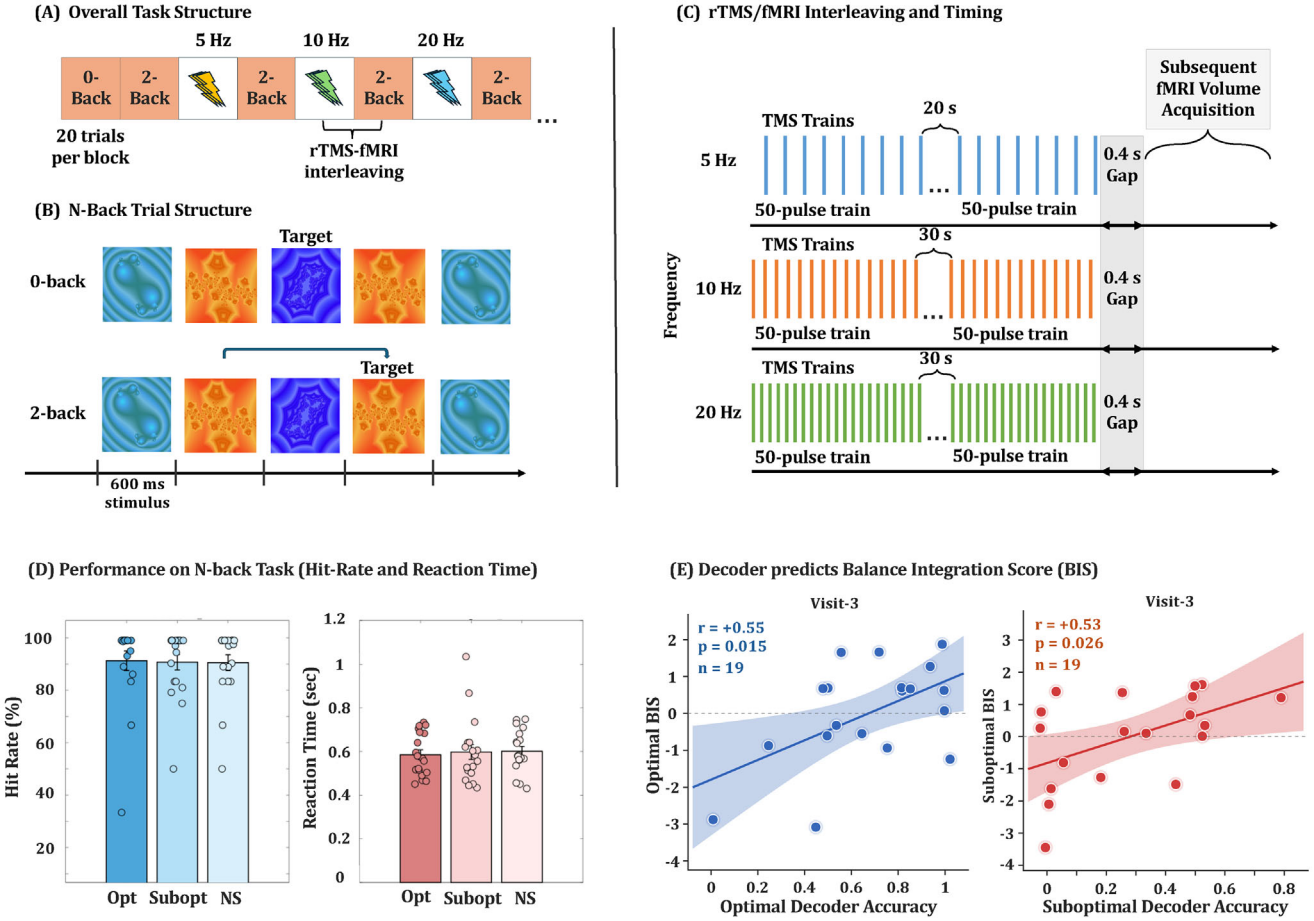
Pre‐Neuromodulation TMS–fMRI Task Design and Behavioral Results. (A) Schematic illustrating the administration of three stimulation frequencies (5, 10, and 20 Hz) during N‐back task blocks at the Pre‐Neuromodulation TMS–fMRI visit. Each 0‐back and 2‐back block comprised a series of 20 trials, during which fMRI was acquired while TMS trains were delivered immediately prior to each task block. (B) Representative examples of 0‐back and 2‐back trial types. In the 0‐back condition, participants pressed a button whenever a dark blue checkered stimulus appeared on screen. In the 2‐back condition, participants responded when the current stimulus matched the one presented two trials earlier, for example the orange checkered stimulus shown in the example. (C) Timing diagram illustrating the TMS–fMRI interleaving protocol. Two consecutive 50‐pulse trains (100 pulses total) at each stimulation frequency were delivered, followed by a 0.4 s gap prior to fMRI acquisition. (D) Group‐average hit rate and reaction time across N‐back conditions for optimal (Opt), suboptimal (Subopt), and no stimulation (NS) frequencies (n = 19). Bars represent the group mean and error bars represent ± 1 SE. Differences across conditions were evaluated using one‐way repeated‐measures ANOVA and were non‐significant for both hit rate and reaction time. (E) Skipped Pearson correlation between decoder output and the Balanced Integration Score (BIS = z[Hit Rate] − z[Reaction Time]). Left panel: optimal stimulation (r(17) = 0.55, p = 0.015, 95% CI [0.124, 0.802]). Right panel: suboptimal stimulation (r(17) = 0.53, p = 0.026, 95% CI [0.095, 0.791]). Each point represents one participant; shaded bands depict 95% bootstrap confidence intervals.

The interleaved TMS–fMRI N‐back task design and associated behavioral results are illustrated in Figure 3. N‐back task blocks were interleaved with rTMS trains at three stimulation frequencies (Figure 3A), with two consecutive 50‐pulse trains (100 pulses total) delivered per frequency immediately prior to each task block, followed by a 400 ms gap before fMRI acquisition (Figure 3C). Each N‐back block comprised 20 trials of either the 0‐back or 2‐back condition (Figure 3B). N‐back performance did not significantly differ across stimulation conditions (optimal, suboptimal, and no stimulation) for either hit rate (F(2, 36) = 0.03, p = 0.975, η^2^_p_ = 0.002) or reaction time (F(2, 36) = 0.71, p = 0.500, η^2^_p_ = 0.038), as shown in Figure 3D.

Despite the absence of group‐level effects, the decoder output significantly correlated with the Balanced Integration Score (BIS), a composite measure computed as z[Hit Rate] − z[Reaction Time] that jointly captures accuracy and response speed, providing a more sensitive index of N‐back task efficiency than either metric alone [34]. This association was observed for both optimal (r(17) = 0.55, p = 0.015, 95% CI = [0.124 0.802]; Figure 3E, left panel) and suboptimal stimulation conditions (r(17) = 0.53, p = 0.026, 95% CI = [0.095 0.791]; Figure 3E, right panel), and was robust to outlier influence as confirmed by the Robust Correlation Toolbox. These findings suggest that decoder output tracked meaningful individual differences in working memory network engagement across stimulation conditions, with the decoder capturing variance in task performance that was not detectable at the group level. Further inspection of individual performance metrics revealed that decoder output also significantly correlated with N‐back hit rate under optimal stimulation (r(17) = 0.56, p = 0.013, 95% CI = [0.140 0.807]). However, this association appeared to be driven by a single data point; excluding this point rendered the correlation non‐significant (r(16) = 0.13, p = 0.60), indicating limited robustness. In contrast, no significant correlation was observed between decoder output and hit rate under suboptimal stimulation (r(17) = 0.35, p = 0.137, 95% CI = [‐0.120 0.696]), and no correlations were observed with reaction time. Together, these results indicate that the composite BIS measure was more reliably predicted by decoder output than either accuracy or reaction time alone, highlighting the value of integrating both performance dimensions when evaluating decoder validity.

### 2.5 Optimal Neuromodulation Enhances Working Memory Performance

**FIGURE 4:**
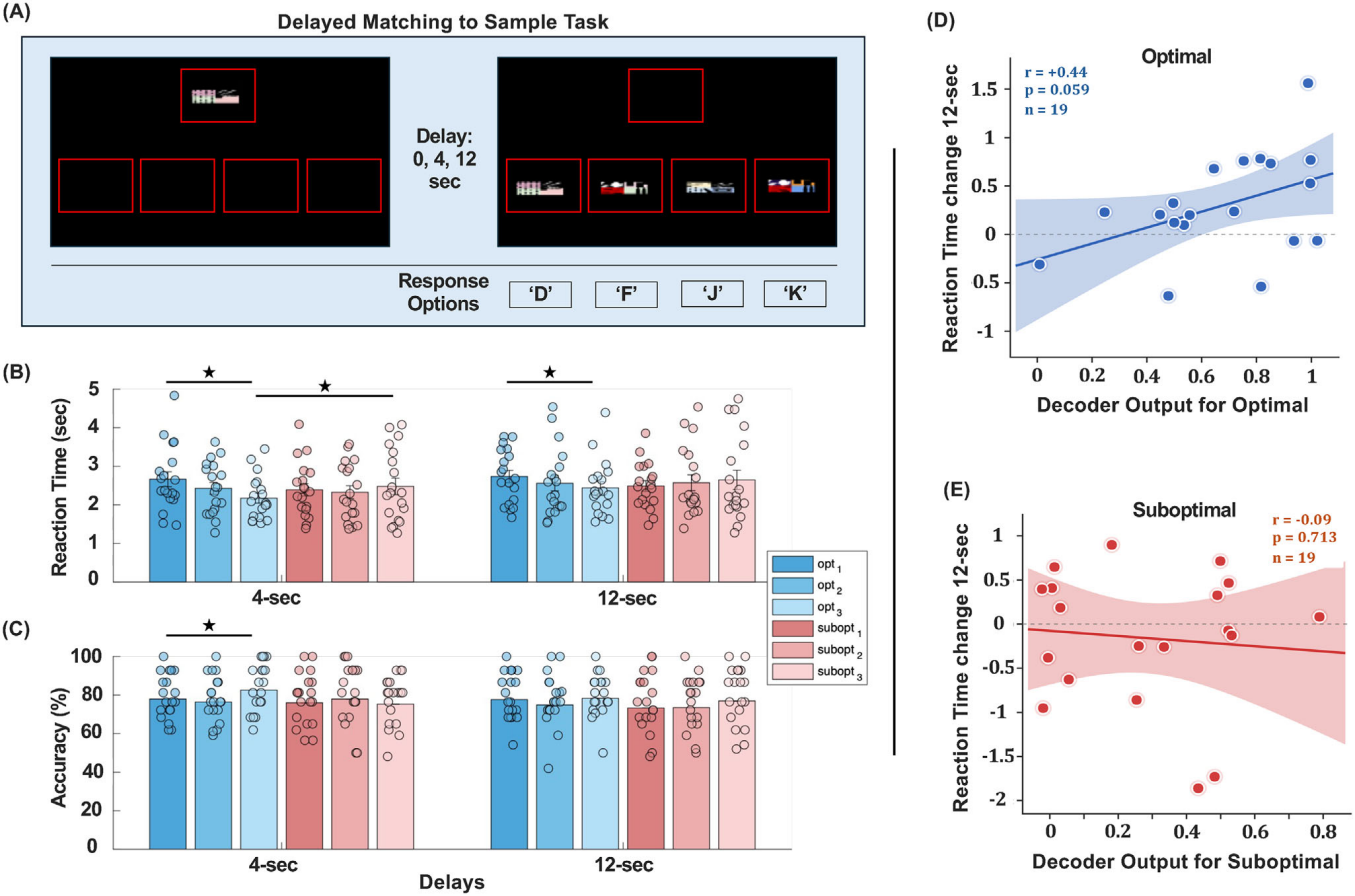
DMTS Task Design and Performance Metrics (A) The figure illustrates the presentation of DMTS stimuli on the screen, followed by a delay of 0, 4, or 12 s, after which participants were required to recall the previously presented item. (B) Reaction time across delay conditions and days (n = 19). Blue bars represent the optimal stimulation condition (three consecutive days, opt₁–opt_3_); red bars represent the suboptimal condition (subopt₁–subopt_3_). Bars represent the group means, and error bars represent ± 1 SE. Data were analysed using three‐way repeated‐measures ANOVA (Stimulation Type × Day × Delay), followed by two‐way ANOVA at each delay and paired‐samples *t*‐tests for significant interactions. Participants were significantly faster on Day 3 than on Day 1 under optimal stimulation at the 4‐s delay (t(18) = 3.31, p = 0.002, Cohen's d = 0.758) and 12‐s delay (t(18) = 2.48, p = 0.023, Cohen's d = 0.568); no such effects were observed under suboptimal stimulation (both p > 0.4). Reaction time at the 4‐s delay on Day 3 was also significantly faster under optimal than suboptimal stimulation (t(18) = 2.24, p = 0.038, Cohen's d = 0.515). (C) Accuracy across delay conditions and days (n = 19). Bar conventions and error bars as in (B). Accuracy at the 4 s delay on Day 3 was significantly higher under optimal than suboptimal stimulation (t(18) = 2.64, p = 0.017, Cohen's d = 0.579). (D, E) Skipped Pearson correlation between pre‐neuromodulation TMS/fMRI decoder output and the change in reaction time at the 12 s delay from Day 1 to Day 3. (D) Optimal condition: r(17) = 0.44, p = 0.059, 95% CI [−0.016, 0.746]. (E) Suboptimal condition: r(17) = −0.09, p = 0.713, 95% CI [−0.523, 0.379]. Shaded bands depict 95% bootstrap confidence intervals. All p‐values are two‐tailed; ^*^ *p* < 0.05,.

Our study employed a delayed matching to sample task (DMTS) during neuromodulation sessions to assess stimulation effects on working memory. In a DMTS task, participants are shown a stimulus, followed by a delay, after which they are presented with multiple stimuli and must identify the original one (a trial is depicted in Figure 4A). The delay period reflects how long participants must retain the information in working memory. Common delay periods used in research, including our study, are 0 s, 4 s, and 12 s. The 0 s delay primarily involves discrimination and encoding processes, while longer delays (4 and 12 s) also engage retention mechanisms. In our study, participants underwent three consecutive optimal neuromodulation sessions and three suboptimal neuromodulation sessions in a randomized order. Importantly, each neuromodulation session involved the administration of 2400 pulses, compared to only 600 pulses during each run of the TMS/fMRI session. Given this difference, we expected to observe a stronger effect on DMTS task performance during neuromodulation sessions. To assess the impact of stimulation on performance in the DMTS task, we performed a repeated measures ANOVA, with neuromodulation stimulation type (optimal vs. suboptimal), stimulation days (1 to 3), and delay (0, 4, or 12 s) as within‐subject variables. Separate tests were conducted for reaction time and accuracy.

For reaction time, we observed a non‐significant trend toward a three‐way interaction (Stimulation Type × Days × Delays) (F(4, 72) = 2.05, p = 0.097, η^2^_p_ = 0.102), a significant two‐way interaction (Stimulation Type × Days) (F(2, 36) = 5.09, p = 0.011, η^2^_p_ = 0.220), and a significant effect for Delays (F(2, 36) = 30.46, p < 0.001, η^2^_p_ = 0.628). Further analysis revealed no interaction between Stimulation Type and Days for the 0 s delay condition (F(2, 36) = 0.60, p = 0.556, η^2^_p_ = 0.032), but a significant effect was found for the 4 s delay (F(2, 36) = 5.60, p = 0.008, η^2^_p_ = 0.237) and the 12 s delay (F(2, 36) = 3.33, p = 0.047, η^2^_p_ = 0.156). Post hoc analysis showed that participants were significantly faster on Day 3 compared to Day 1 during optimal neuromodulation for both the 4 s delay (t(18) = 3.31, p = 0.002, Cohen's d = 0.758) and the 12 s delay (t(18) = 2.48, p = 0.023, Cohen's d = 0.568). However, no such effects were observed under the suboptimal stimulation for either a 4 s (t(18) = 0.55, p = 0.586, Cohen's d = 0.126) or 12‐second (t(18) = 0.86, p = 0.404, Cohen's d = 0.196) delay condition. Additionally, the reaction time on a 4 s delay on Day 3 of optimal stimulation was significantly better than the 4 s delay under suboptimal stimulation on Day 3 (t(18) = 2.24, p = 0.038, Cohen's d = 0.515). Figure 4B illustrates the significant effects observed for reaction time.

For accuracy, there was no significant three‐way interaction (Stimulation Type × Days × Delays) (F(4, 72) = 0.63, p = 0.644, η^2^_p_ = 0.034), nor was there a significant two‐way interaction for Stimulation Type × Delays (F(2, 36) = 0.91, p = 0.413, η^2^_p_ = 0.048) or Stimulation Type × Days (F(2, 36) = 0.09, p = 0.919, η^2^_p_ = 0.005). Only the main effect for Delays was significant (F(2, 36) = 11.360, p < 0.001, η^2^_p_ = 0.387). To explore whether the improvement in reaction time came at the expense of accuracy, paired *t*‐tests were conducted on the variables that showed significant differences in reaction time. These tests indicated that participants were slightly more accurate for the 4 s delay on Day 3 of optimal stimulation compared to suboptimal stimulation (t(18) = 2.64, p = 0.017, Cohen's d = 0.579), as shown in Figure 4C. No other comparisons reached statistical significance for either optimal or suboptimal conditions. We thus did not find evidence for a speed/accuracy trade‐off in explaining the reaction time improvements specific to optimal stimulation neuromodulation. Overall, the results indicated that during optimal neuromodulation sessions, participants responded more quickly when recalling information after longer delays (4 and 12 s), which are known to engage retention mechanisms, without a reduction in accuracy. For more details on the performance metrics, including the 0‐delay condition, see Figure S4.

### 2.6 Brain Readouts From the Decoder Predicted Performance in the Working Memory Task

We further investigated whether the decoder outputs from the TMS/fMRI sessions were related to behavioral performance improvements on the DMTS task. While not statistically significant, we found that decoder output during pre‐neuromodulation TMS‐fMRI session showed a correlation in the positive direction with reaction time changes in the 12‐delay condition from Day 1 to Day 3 (r = 0.44, p = 0.059, 95% CI = [−0.016 0.746]), as shown in Figure 4D. This relationship was not observed for the suboptimal stimulation when we correlated the suboptimal decoder output with suboptimal changes in the 12 s delay condition (r = ‐0.09, p = 0.713, 95% CI = [−0.523 0.379]), as shown in Figure 4E.

### 2.7 Optimal Frequency Stimulation Enhances Performance, Irrespective of Specific Frequency Band

During neuromodulation training sessions, each participant received stimulation at two (optimal and suboptimal) out of three possible frequencies (5, 10, or 20 Hz) at their individualized target locations. To examine the possibility that a specific frequency was optimal across the participant cohort, we employed an exploratory Linear Mixed‐Effects (LME) Model, treating Frequency as a fixed effect and Participant as a random effect to account for the unbalanced design. The three‐way interaction between stimulation frequency, delay, and stimulation day on reaction time for the DMTS task was not statistically significant (F(8, 264.6) = 1.41, p = 0.192, Cohen's f^2^ = 0.043), suggesting that the combined influence of frequency and delay on reaction time did not differ across stimulation days. Similarly, for accuracy, the three‐way interaction between frequency, delay, and visit number for the DMTS task was not significant (F(8, 251.3) = 1.55, p = 0.142, Cohen's f^2^ = 0.049). These findings suggest that frequency alone did not determine stimulation effects; rather, it was the combination of frequency and target. The mean reaction time and accuracy with standard errors are provided in the supplementary materials in Figures S5 and S6, respectively. Table S2 provides information about the frequencies assigned as optimal and suboptimal for each of the participant.

### 2.8 Stimulation Effects Were Specific to the Working Memory Task

**FIGURE 5:**
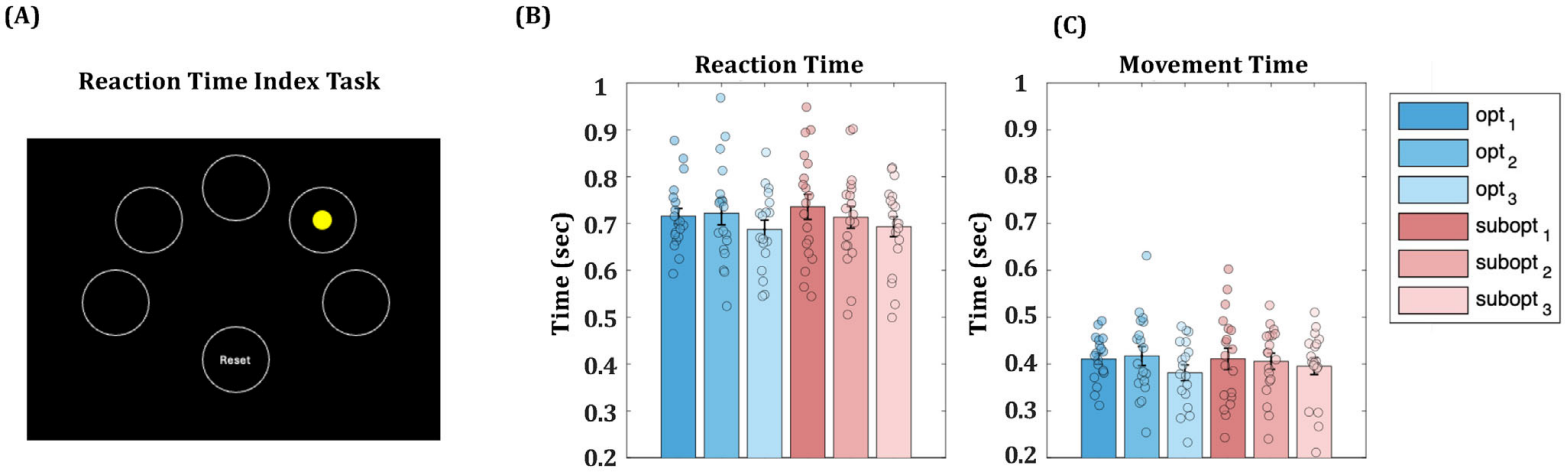
Reaction Time Index Task and Performance Measures. (A) Schematic of an RTI trial. Participants held down the reset button at the start of each trial. When the yellow circle appeared on one of the five target circles, they moved their finger to the indicated target button. Reaction time was defined as the interval from yellow‐circle onset to release of the reset button; movement time was defined as the interval from reset‐button release to target‐button press. Group‐average reaction time (B) and movement time (C) across optimal and suboptimal stimulation conditions on each of the three training days (n = 19). Blue bars represent the optimal condition (opt₁–opt_3_); red bars represent the suboptimal condition (subopt₁–subopt_3_). Bars represent the group means, and error bars represent ± 1 SE. Data were analysed with two‐way repeated‐measures ANOVA (Stimulation Type × Day). No significant Stimulation Type × Day interaction was observed for reaction time (F(2, 36) = 0.84, p = 0.439, η^2^_p_ = 0.045) or movement time (F(2, 36) = 0.59, p = 0.563, η^2^_p_ = 0.032).

During the neuromodulation sessions, a Reaction Time Index (RTI) task was administered to assess processing speed and motor reaction time. In this task, participants were instructed to keep the ‘Reset’ button pressed and press a second target button when the target appeared in a yellow circle (a trial is shown in Figure 5A). Reaction time was defined as the duration from target presentation to button release, while movement time referred to the duration from ‘Reset’ button release to pressing the target button.

The RTI task served as a control to isolate the effects of stimulation on working memory, ensuring that any observed effects were specific to working memory processes rather than general processing speed. A repeated measures ANOVA was conducted with neuromodulation condition (optimal vs suboptimal) and days (1 to 3) as within‐subject variables. No interaction between neuromodulation condition and days was observed for either average reaction time (F(2, 36) = 0.84, p = 0.439, η^2^_p_ = 0.045) or movement time (F(2, 36) = 0.59, p = 0.563, η^2^_p_ = 0.032). These results support the hypothesis that stimulation influenced only working memory or memory retention processes, while processing speed in a non‐working memory‐related task remained unaffected. The results from the RTI task are shown in Figure 5B,C.

### 2.9 Decoder Brain Readouts and Behavioral Performance After Neuromodulation Training

After completing the optimal and suboptimal neuromodulation sessions, TMS‐fMRI sessions were conducted to assess the network engagement based on decoder accuracy and N‐back task performance. Both optimal and suboptimal training sessions were followed by TMS‐fMRI sessions involving both optimal and suboptimal frequency stimulations during the N‐Back task. The order of frequency administration is provided in Table S3. We expected that network engagement would be improved following neuromodulation training, particularly during optimal frequency stimulation, and that will reflect in improved accuracy in decoder output.

**FIGURE 6:**
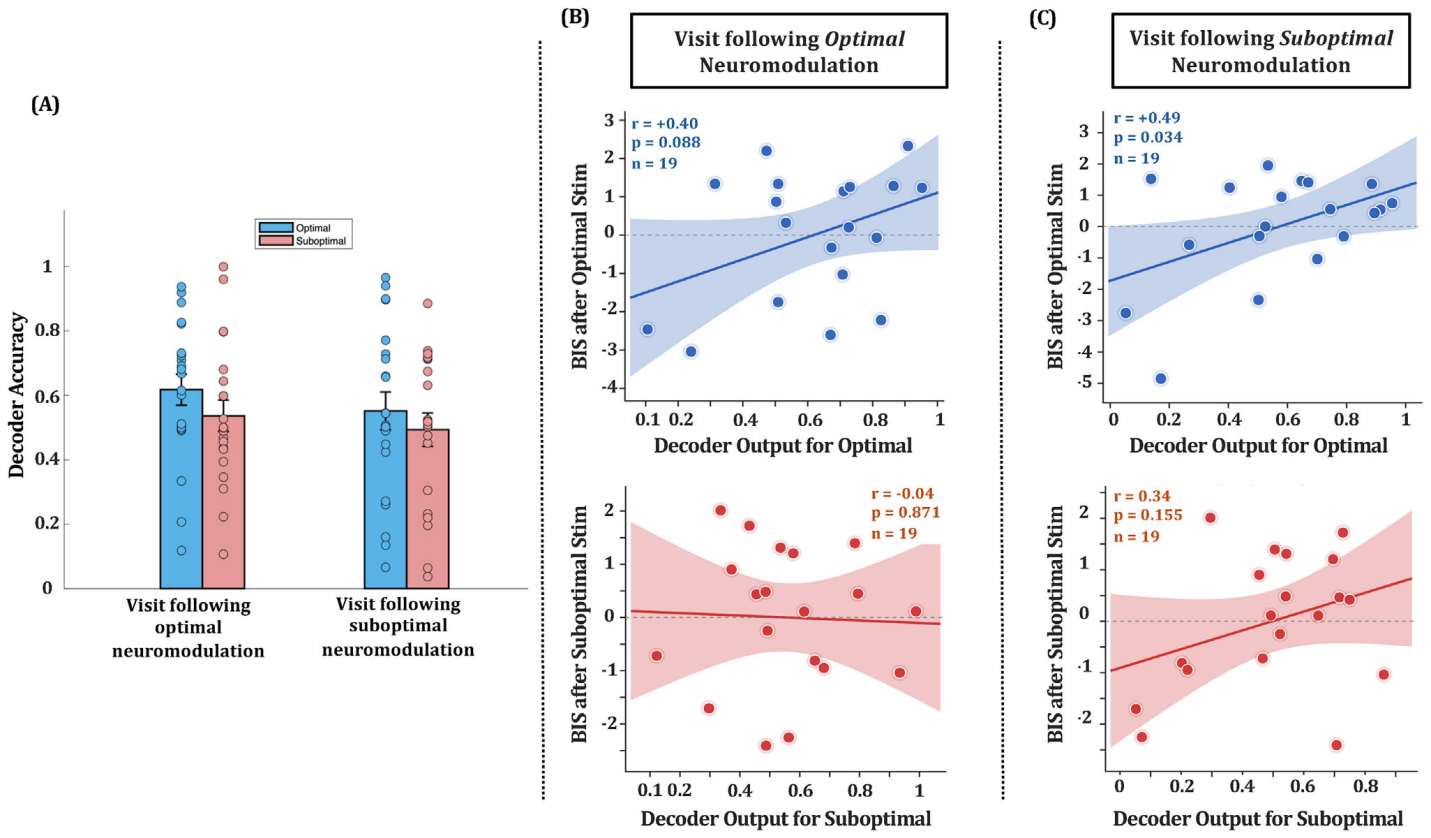
Decoder Output and Behavioral Performance after Neuromodulation Training. (A) Decoder output during post‐neuromodulation TMS/fMRI visits (n = 19). The left bars shows the visit following optimal neuromodulation and the right bars show the visit following suboptimal neuromodulation. Blue bars represent optimal stimulation and red bars represent suboptimal stimulation; both frequencies were administered at each post‐neuromodulation visit. Bars represent the group mean and error bars represent ± 1 SE. Data were analysed with two‐way repeated‐measures ANOVA (Decoder Output × Neuromodulation Session Type), which yielded no significant interaction (B) Skipped Pearson correlations between decoder output and BIS at the TMS/fMRI visit following optimal neuromodulation. Top panel: optimal stimulation, r(17) = 0.40, p = 0.088, 95% CI [0.065, 0.723]. Bottom panel: suboptimal stimulation, r(17) = −0.04, p = 0.871, 95% CI [−0.497, 0.408]. (C) Skipped Pearson correlations between decoder output and BIS at the TMS/fMRI visit following suboptimal neuromodulation. Top panel: optimal stimulation, r(17) = 0.49, p = 0.034, 95% CI [0.043, 0.771]. Bottom panel: suboptimal stimulation, r(17) = 0.34, p = 0.155, 95% CI [−0.136, 0.688]. Each point represents one participant; shaded bands depict 95% bootstrap confidence intervals. All p‐values are two‐tailed.

We conducted an interaction analysis using ANOVA to assess how decoder performance during the TMS/fMRI visits differed after these sessions. There was no significant interaction observed between the decoder output (optimal vs. suboptimal) and the type of neuromodulation session, F(1, 18) = 0.03, p = 0.857, η^2^_p_ = 0.002. Additionally, there was a marginal effect of stimulation, F(1, 18) = 3.70, p = 0.070, η^2^_p_ = 0.171. The decoder output for the optimal condition was slightly better than for the suboptimal condition, as illustrated in Figure 6A. Furthermore, there was no interaction observed in N‐back hit rate between stimulation type (optimal, suboptimal, or no stimulation) and the type of preceding neuromodulation training (optimal vs. suboptimal), F(2, 36) = 0.01, p = 0.988, η^2^_p_ < 0.001). Similarly, there was no significant interaction effect between neuromodulation training and stimulation type on reaction time, F(2, 36) = 0.31, p = 0.737, η^2^_p_ = 0.017.

We further examined the relationship between decoder output and N‐back task performance across post‐neuromodulation TMS‐fMRI sessions. Following optimal neuromodulation, decoder output for optimal stimulation showed a positive but non‐significant trend with BIS (r = 0.40, p = 0.088, 95% CI = [0.065 0.723]; Figure 6B, top panel), while no such trend was observed for suboptimal stimulation (r = −0.04, p = 0.871, 95% CI = [−0.497 0.408]; Figure 6B, bottom panel). Furthermore, N‐back hit rate was significantly correlated with decoder output for optimal stimulation (r = 0.48, p = 0.037, 95% CI = [0.034 0.767]), but not for suboptimal stimulation (r = −0.01, p = 0.951, 95% CI = [−0.465 0.442]). Similarly, following suboptimal neuromodulation, decoder output for optimal stimulation remained significantly associated with both BIS (r = 0.49, p = 0.034, 95% CI = [0.043 0.771]; Figure 6C, top panel) and N‐back hit rate under optimal stimulation (r = 0.55, p = 0.016, 95% CI = [0.122 0.801]). In contrast, decoder output for suboptimal stimulation did not show a significant association with BIS (r = 0.34, p = 0.155, 95% CI = [−0.136 0.688]; Figure 6C, bottom panel), although it showed a strong correlation with hit rate under suboptimal conditions (r = 0.74, *p* < 0.001, 95% CI = [0.430 0.894]). Overall, the decoder–behavior associations observed during the pre‐neuromodulation TMS–fMRI session were sustained across both post‐neuromodulation sessions, suggesting that the relationship between working memory network engagement and behavioral performance remained stable following neuromodulation training.

## 3 Discussion

Attempts to enhance working memory with TMS have yielded inconsistent results, in part because stimulation studies have often overlooked individual neural dynamics. In this study, we developed a personalized, decoder‐guided neuromodulation approach that optimizes both stimulation site and frequency. By integrating PFNs derived from fMRI data with a neural network‐based decoding model, we enabled adaptive, state‐dependent stimulation. This approach allowed us to identify optimal and suboptimal stimulation frequencies based on decoder predictions and behavioral performance. The identified frequencies were then evaluated across multiple neuromodulation sessions during which participants performed the DMTS working memory task and a control task assessing processing speed. Critically, optimal stimulation produced significantly faster responses in a DMTS task at 4‐ and 12 s delays, without compromising accuracy. These effects were specific to working memory, as stimulation did not affect control task performance, nor were they explained by any specific frequency alone. Together, these findings demonstrate that personalized, network‐based neuromodulation can enhance human working memory and provide a brain decoding framework for neuromodulation approaches.

The primary objective of this study was to develop a brain decoding framework that leverages recent advances in PFNs derived from fMRI, combined with deep learning techniques, for adaptive brain stimulation aimed at enhancing working memory. Utilizing functional profiles extracted from working memory fMRI data based on PFNs as input features, our deep learning models learned precise mappings between dynamic brain states and stimulation‐induced brain changes [32, 35]. Importantly, we incorporated interpretable deep learning methods to rigorously identify the most informative and redundant components within our models. This approach facilitated individualized, precise targeting for TMS with concurrent fMRI, enabling real‐time adaptive adjustment of stimulation parameters. Moreover, unlike most traditional network analysis techniques that rely on group‐level FCN decompositions or static brain parcellations, our framework captured the dynamic variations of PFNs across both resting and task‐evoked brain states [36, 37, 38, 39, 40, 41, 42]. This integration of resting‐state and task‐based data allowed for more accurate identification of stimulation sites tailored to individual brain dynamics. Overall, this work introduces a methodological framework for personalized and adaptive brain stimulation, integrating PFN‐based fMRI analysis, deep learning with LSTM RNNs, and concurrent TMS/fMRI to enhance working memory performance in healthy individuals.

During the neuromodulation training involving a working memory task, where optimal versus suboptimal frequencies were tested using our approach, we found that the optimal stimulation only influenced DMTS performance in 4 and 12 s delay trials. The DMTS captures multiple aspects of cognition, including discrimination, encoding, and retention [43]. Discrimination involves recognizing the presented information, encoding refers to converting this information into an internal representation, and retention is the ability to maintain it over time. In the 0‐delay condition, encoding and retention mechanisms are not engaged since there is no delay between the presentation of the to‐be‐remembered trial and the recall trial. However, when the delay period exceeds 0 s, these mechanisms come into play. The fact that we did not observe any stimulation effect on the 0 s condition suggests that stimulation had no influence on the discrimination, and it only influenced mechanisms engaged in retention of working memory, with only 4 s and 12 s showing improved reaction time, particularly on the final day of optimal neuromodulation. Importantly, the accuracy in the optimal neuromodulation sessions was also better than that of the suboptimal neuromodulation sessions. Furthermore, the RTI task that assesses speed in the non‐working memory‐related task was not influenced by the type of stimulation, suggesting that only working memory‐related mechanisms were modulated by stimulation. In addition, we examined whether there were frequency‐specific effects by comparing performance under 5, 10, and 20 Hz stimulation during the DMTS task. The analysis did not reveal any significant effects related to stimulation frequency as an independent factor. While prior research has linked specific frequencies to distinct neurobiological effects, we observed that the combination of individualized targeting and frequency is what ultimately drives neuromodulatory optimization. By tailoring stimulation to each participant's unique brain target, we likely engaged diverse network dynamics, leading to target‐dependent responses to frequency.

Surprisingly, no stimulation effect was evident in the N‐back task during the Pre‐neuromodulation TMS/fMRI sessions. Although this finding is unexpected, several underlying factors could plausibly account for this, including differences in TMS dosage and a potential ceiling effect in the N‐back task. During the TMS/fMRI sessions, only 600 pulses of rTMS were administered in one run, compared to the 2400 pulses delivered in each neuromodulation session. The reduced dosage in TMS/fMRI sessions was due to time constraints, as the primary goal of those sessions was to engage the working memory networks. This lower stimulation dose may have contributed to the lack of observable effects on N‐back performance. Moreover, participants performed exceptionally well during the N‐back task, with elevated hit rates suggesting a ceiling effect, leaving little room for measurable improvement. These factors collectively may have contributed to the absence of a detectable stimulation effect during the N‐back task. Importantly, despite the null group‐level effects, decoder output significantly tracked the Balanced Integration Score (BIS), a composite index capturing the trade‐off between accuracy and reaction time. This suggests that the decoder is sensitive to latent efficiency‐related differences in working memory performance. Notably, while decoder output showed a significant association with hit rate under optimal stimulation, this effect was not robust and did not survive after outlier removal. In contrast, no significant relationship was observed between decoder output and hit rate under suboptimal stimulation. Together, BIS appears to provide a more sensitive and behaviorally informative measure of task efficiency, aligning more closely with underlying network dynamics targeted by stimulation.

An interesting and unexpected finding from our study was that the decoder performance improved for suboptimal stimulation after neuromodulation training, whereas it plateaued for optimal stimulation. One speculative but theoretically motivated interpretation of this unexpected finding can be explained through the interplay of Hebbian and Homeostatic plasticity. Hebbian plasticity strengthens connections between neurons as they fire together, while homeostatic plasticity prevents excessive synaptic changes [44]. During the pre‐neuromodulation TMS/fMRI visit, the brain is responsive, and the optimal stimulation possibly results in Hebbian‐like plasticity by engaging the working memory network that reflects in higher decoder accuracy. However, once this optimized state was reached, the homeostatic mechanisms may have prevented further engagement of working memory networks. In contrast, due to low plastic gain during suboptimal stimulation at Pre‐Neuromodulation TMS/fMRI visit, the threshold for further plasticity may be lower, resulting in greater plastic shifts when tested again after neuromodulation training. In addition, there was no correlation between decoder output for suboptimal stimulation and N‐back performance metrics during suboptimal stimulation after optimal neuromodulation. This absence may indicate that once the brain is brought into a more efficient or selective state through optimal neuromodulation, it becomes less responsive to suboptimal stimulation frequencies, potentially reflecting a tuning of functional networks to optimal frequency. We acknowledge that these interpretations regarding what the decoder may be capturing are speculative, and we currently lack strong empirical evidence to fully support the proposed models. Nevertheless, this remains a promising direction for future investigation. It is worth considering that while stimulation frequencies were selected during the N‐back task, it is possible that these frequencies reflect alignment with task‐specific network dynamics rather than stable, intrinsic, individualised parameters. The observation that stimulation at the selected frequencies improved performance on a distinct working memory paradigm (DMTS) provides partial support for generalization, though both tasks recruit overlapping frontoparietal networks, and cross‐task transfer within the working memory domain does not fully exclude a task‐dependent account. Future work examining whether such frequency preferences remain consistent across cognitively distinct task states, including non‐working memory paradigms, will be important for establishing the degree to which they represent trait‐like stimulation properties.

While this study introduces an innovative approach to personalizing brain stimulation using real‐time neural readouts, it has several limitations that warrant consideration. First, although the decoder output during informed loop correlated with behavioral performance, it did not reliably predict the optimal and suboptimal stimulation frequencies after the random loop at the individual level on the day of the experiment. Specifically, in 9 out of 19 participants (47%), the decoder did not produce a sufficiently discriminative real‐time output to meet the predefined decision criterion, necessitating a hierarchical behavioral fallback procedure whereby hit rate and reaction time guided frequency selection. This relatively high fallback rate likely reflects the operational constraints inherent to simultaneous TMS–fMRI, including limited decision time windows and the challenge of achieving robust signal contrast between cognitive states in real time. Consistent with this interpretation, participants in whom the decoder successfully classified frequencies exhibited a higher temporal contrast‐to‐noise ratio (tCNR) of WM‐relevant functional networks than those requiring the behavioral fallback, suggesting that inter‐individual differences in neural signal quality contributed to decoder reliability. In addition, the decoder consistently discriminated between optimal and suboptimal stimulation across all fine‐tuned versions, with larger output differences observed for optimal stimulation in each case. The statistical reliability improved in later versions, likely reflecting model refinement. The consistency of this effect across independently fine‐tuned versions suggests that the observed discrimination is robust and not driven by any single stage of decoder optimization. In light of the above factors, we acknowledge that the current implementation represents a proof‐of‐concept for an adaptive and personalized neuromodulation approach rather than a fully realised system, and future work should prioritise improving real‐time decoding robustness through enhanced preprocessing pipelines, adaptive thresholding strategies, and fine‐tuning on larger, study‐specific datasets.

In addition, the final analytical sample of N = 19 was smaller than the planned enrollment target due to resource and time constraints. Nevertheless, the crossover design, with three neuromodulation sessions per condition, reduces within‐subject variance and provides greater sensitivity than a simple paired comparison. Although the achieved sample fell below the planned enrollment, post hoc power estimates for the primary behavioral outcome (i.e., the DMTS task) showed effect sizes of Cohen's d = 0.758 (4 s delay) and Cohen's d = 0.568 (12 s delay), indicating that the study retained sufficient statistical power at N = 19. Furthermore, the decoder‐classified difference in working memory network engagement between optimal and suboptimal stimulation yielded a large effect (Cohen's d = 1.56), suggesting that the sample was adequate to detect stimulation‐induced neural changes. Moreover, analytical samples of N = 15–25 are normative for intensive multi‐session TMS–fMRI studies employing personalized targeting [45, 46]. Nonetheless, the modest sample size may limit the precision of secondary behavioral effect size estimates and the generalizability of findings to broader populations, and replication in larger confirmatory trials is warranted. Furthermore, the lack of a control neuromodulation condition limits our ability to directly assess how optimal and suboptimal training compares to a true baseline. It should be noted that % maximum stimulator output (MSO) for TMS was not systematically recorded during data collection. Future studies employing this distance‐adjusted rMT approach should record and report (%MSO) alongside individualised threshold values to facilitate cross‐study comparisons. Finally, the study was conducted exclusively with healthy participants, so caution is needed when extending these results to clinical populations, whose baseline neural activity and responsiveness to TMS may differ significantly. Expanding the sample to include a larger and more diverse group, particularly individuals with clinical conditions, will be essential for evaluating the broader applicability and effectiveness of this approach.

## 4 Conclusion

In summary, we show that a precision neuromodulation approach, tailored to individual brain structure and neural activity, improves working memory performance. Beyond the behavioral effect, a key contribution of this work lies in the methodological framework, demonstrating that stimulation parameters can be informed by subject‐specific neural signatures and task‐relevant brain states. This provides a step toward more principled and individualized neuromodulation strategies. While promising, these findings should be interpreted in light of important limitations. The durability of the observed effects, their generalizability to clinical populations, and replication in larger and more diverse samples remain to be established. Future work will be needed to determine whether this approach can be extended across cognitive domains and translated into clinically meaningful interventions.

## 5 Materials and Methods

### 5.1 Subject Details

A total of 27 participants were recruited for this study. A formal a priori power analysis was conducted to estimate the sample size. Based on pilot data indicating a mean (SD) change in working memory performance (d′) of approximately 0.8 (0.87), we estimated that N = 40 enrolled participants would provide 80% power to detect a mean change of 0.4 (Cohen's d ≈ 0.46) and 90% power to detect a change of 0.46 (Cohen's d ≈ 0.53). This calculation was based on a single‐session paired comparison and therefore represents a loose estimate given the repeated‐measures crossover design of the study, in which three neuromodulation sessions per condition per participant reduce within‐subject variance and afford greater effective power than the enrolled sample size alone would suggest. Data collection was curtailed due to funding constraints, with 27 participants enrolled. Of the 27 enrolled participants, 23 completed all 11 study sessions. Three participants were excluded due to technical issues, such as hardware failure during TMS/fMRI acquisition and software glitches resulting in incomplete data files in at least one of the 11 sessions. An additional participant was excluded due to an apparent misunderstanding of the task instructions and unreliable performance. Analytical samples of N = 15–25 are well within the normative range for intensive multi‐session TMS–fMRI studies employing personalized targeting approaches, reflecting the substantial per‐participant burden of such paradigms. The final sample for analysis included 19 participants (N = 9 males). Participants were healthy right‐handed adults (ages: 18–38, mean age = 25.43 ± 5.71 years) with no current or prior psychiatric or neurological disorders. The study was approved by the University of Pennsylvania IRB (IRB Protocol #832891). All participants gave written informed consent after receiving a complete description of the study according to the Declaration of Helsinki.

### 5.2 Study Design

Participants attended two baseline assessment visits. During Visit 1, participants completed questionnaires assessing demographic information and eligibility, including the MRI Safety Form and the Treatment‐Associated Side Effects Scale (TASS). A structured clinical interview using the Structured Clinical Interview for DSM‐5 was administered by a trained interviewer to confirm diagnostic and eligibility criteria. Participants who met the inclusion criteria then proceeded to Visit 2, during which a comprehensive battery of self‐report measures was collected to assess mood, affect, psychiatric symptoms, sleep, stress, and related factors. These included measures of acute mood and affect (PANAS), anhedonia (SHAPS), worry (PSWQ), sleep quality (PSQI, ISI), trauma exposure (SLESQ, CTQ), depressive symptoms (PHQ‐9, PDQ‐D5, DASS‐21), perceived social support (MSPSS), and smoking‐related variables (baseline smoking behavior and smoking urges). Eligible participants subsequently completed a baseline MRI session (approximately 1 h) on the same day, which included structural imaging and functional MRI acquired during an N‐back working memory task and at rest.

An approximately two‐week period was allocated to compute individualised stimulation targets and to derive subject‐specific brain state decoders prior to the initial TMS/fMRI session. During the Pre‐neuromodulation TMS/fMRI session (Visit 3), rTMS at multiple frequencies was interleaved with N‐back blocks. Decoder output reflecting working memory network engagement and concurrent behavioral performance was used to classify each stimulation frequency as optimal or suboptimal for each individual participant. After Pre‐neuromodulation TMS/fMRI session, participants entered a randomized crossover neuromodulation phase from the very next day. Each condition consisted of three consecutive daily sessions of rTMS neuromodulation using either the individually identified optimal or suboptimal stimulation frequency, followed by an additional TMS/fMRI session. The two frequency conditions were administered in separate weeks, with a minimum one‐week washout period between conditions to allow sufficient dissipation of any residual stimulation effects (six neuromodulation sessions total). Condition order was randomized across participants.

During each neuromodulation session, participants performed two tasks following rTMS: a working memory task and a control task. The working memory task was a delayed matching‐to‐sample (DMTS) task, in which participants retained a visual stimulus across a delay period and were subsequently tested on recognition. This task differed deliberately from the N‐back task used during the TMS/fMRI sessions, in order to assess whether stimulation effects generalize across working memory paradigms. The control task assessed processing speed and was included to verify that any observed changes in working memory performance were specifically attributable to stimulation effects on working memory processes rather than reflecting non‐specific changes in arousal or motor response. Post‐neuromodulation TMS/fMRI sessions (Visits 7 and 11) were conducted immediately following neuromodulation days to assess network‐level and behavioral effects of the neuromodulation training. The details of the visit are provided in Figure 1.

### 5.3 Cognitive Assessments

#### 5.3.1 N‐Back Task

The working memory task conducted during the TMS/fMRI was an N‐back task consisting of a 0‐back condition and a 2‐back condition. We adopted a fractal N‐back version of the working memory task paradigm from [47], which was originally developed to investigate executive system maturation during adolescence. Each run comprised 10 blocks, with each block containing 20 trials, during which fractal images were presented in a randomized order. Fractal stimuli were displayed for 600 ms, followed by an interstimulus interval of 2400 ms. In the 0‐back condition, participants were instructed to respond when the displayed fractal stimulus matched the pre‐specified target stimulus. In the 2‐back condition, participants were required to indicate when the current stimulus matched the fractal image shown two trials prior. Between 2‐back blocks, TMS was delivered in the form of 50‐pulse trains at 5, 10, or 20 Hz. Importantly, the onset of stimulation was temporally adjusted across frequencies such that the final TMS pulse occurred 0.4 s before the onset of the subsequent fMRI volume acquisition. This ensured a constant 0.4 s interval between the end of stimulation and fMRI readout across all frequency conditions, allowing for comparable hemodynamic measurement timing. The Task design is shown in Figure 3B. To provide a more comprehensive validation of decoder performance, we computed the Balanced Integration Score (BIS = z[Hit Rate] − z[Reaction Time]) as a composite behavioral metric capturing both accuracy and response speed while accounting for the speed–accuracy trade‐off [34]. BIS was used in addition to hit rate and reaction time as a validation metric, given that the N‐back decoder was designed to distinguish 2‐back from 0‐back conditions, a distinction that can manifest as improvements in either accuracy, response time, or both.

#### 5.3.2 DMTS Task

The Delayed Matching to Sample (DMTS) task, adopted from the Cambridge Neuropsychological Test Battery (CANTAB; Cambridge Cognition, [48]). During the task, a visual target pattern was presented for 3 s. After the target pattern disappeared, four comparison patterns were displayed following a delay of 0, 4, or 12 s. Reaction time and accuracy during the trials were used as outcome measures. Each delay condition included 13 trials, which were randomized across the different delay conditions. An example of a task trial is illustrated in Figure 4A.

#### 5.3.3 RTI Task

The Reaction Time Index (RTI) task was used to assess motor and mental reaction speeds following neuromodulation. This task was also adopted from the Cambridge Neuropsychological Test Battery (CANTAB; Cambridge Cognition, [48]). The task featured 6 circles on a black screen. Participants were instructed to select and hold the bottom circle using their mouse. A yellow dot flashed on one of the remaining 5 circles. Participants were required to move their mouse and click on the indicated target. A total of 40 trials were presented. A trial example is shown in Figure 5A.

### 5.4 MRI Acquisition

MRI data were acquired using a 3 Tesla Siemens Prisma scanner. Baseline scans (T1‐weighted and resting‐state fMRI) were conducted with a standard 32‐channel head coil (Erlangen, Germany). For interleaved TMS/fMRI scans, a custom “birdcage” head coil was used to accommodate the MRI‐compatible TMS coil (RAPID quad T/R single channel; Rimpar, Germany) and a custom‐built TMS coil holder. Two baseline resting‐state fMRI scans were obtained with opposite phase encoding directions (A>>P and P>>A) using the following parameters: TR = 1355 ms, TE = 32 ms, FA = 68°, FOV = 216 mm, voxel size = 2.4 × 2.4 × 2.4 mm, 72 interleaved axial slices (no gap), and 640 volumes. Participants were instructed to keep their eyes open, remain still, and fixate on a central cross. High‐resolution structural images were acquired using a multi‐echo T1‐weighted MPR sequence with the following parameters: TR = 2400 ms, TI = 1060 ms, TE = 2.24 ms, FA = 8°, voxel size = 0.8 × 0.8 × 0.8 mm, FOV = 256 mm, PAT mode = GRAPPA, and 208 slices.

### 5.5 Simultaneous TMS/fMRI Session

TMS was administered within the MRI bore using an MRI‐compatible Magventure MRI‐B91 air‐cooled coil connected to a MagPro X100 stimulator (Magventure, Farum, Denmark). Stimulation sites were marked on a Lycra swim cap on a participant's head using neuronavigation immediately before the MRI session. The coil was positioned inside the MRI head coil, with the cable routed through the bore to generate a posterior‐to‐anterior induced current. Resting motor threshold (rMT) was determined in the MRI room by visually observing motor activity in the abductor pollicis brevis of the right hand. This accounted for potential influences from filters, cable length, and magnetic field interference. The rMT was defined as the minimum power required to elicit a motor reaction in 5 out of 10 stimulations. Importantly, electromyography (EMG)‐based threshold determination, which is typically used in TMS studies, was not feasible in the present study, as electromagnetic interference from the MRI scanner environment precludes EMG recording. While visual observation represents the most practical alternative under these constraints, it is acknowledged that this method typically yields higher rMT estimates than EMG‐based approaches, which may have resulted in marginally elevated stimulation intensities relative to EMG‐calibrated studies and introduced a degree of additional inter‐individual variability in effective stimulation dose. In addition, because non‐motor cortical targets do not provide a direct functional readout for intensity adjustment, the motor threshold was further adjusted according to the relative scalp‐to‐cortex distance between the motor cortex and the stimulation target. This adjustment was applied to account for regional variation in coil‐to‐cortex distance, which is known to influence the effective strength of cortical stimulation [49, 50]. The motor threshold was adjusted according to the scalp‐to‐cortex distance [50] using the following formula:

$$djMT=rMT+2.8\left(D_{siteX}-D_{M1}\right)$$

D_siteX_ is the cortical distance to the target, and D_M1_ is the cortical distance to the motor cortex. Stimulation intensity for TMS was then set to 120% of adjusted MT. MRI volume acquisition (TR = 2000 ms, TE = 30 ms, FA = 75°, FOV = 192 mm, voxel size = 3 × 3 × 4 mm, 32 interleaved axial slices, 625 volumes) was synchronized with the working memory task and TMS pulses using TTL triggers sent via a parallel port from a Windows PC running PsychoPy (Version 2023.1.3). A 400 ms gap was inserted between the rTMS trains and subsequent fMRI acquisition to prevent contamination of the acquired volumes. For each stimulation frequency, two consecutive 50‐pulse trains (100 pulses total) were delivered immediately prior to each N‐back task block during which fMRI was acquired, as shown in Figure 3C. Data from runs with excessive motion (relative motion > 0.2 mm) were excluded from further analysis. A total of 600 pulses were delivered during each run of the MRI volume acquisition while the participant was performing the working memory task. The order of frequencies administered was dependent on the decoder version. Results from the first TMS/fMRI session determined the frequency of TMS administered in the rest of the study sessions. The three frequencies used were 5 Hz, 10 Hz, and 20 Hz. All frequencies were administered in 50 pulse trains with a 20 s inter‐train interval for the 5 Hz frequency, and a 30 s inter‐train interval for both the 10 Hz and 20 Hz frequencies.

### 5.6 Neuromodulation Sessions

Following the Pre‐Neuromodulation TMS/fMRI session, two frequencies (optimal and suboptimal) were selected for each participant based on decoder‐derived network engagement or behavioral performance. Within each neuromodulation arm, the same individually selected frequency was administered across all three consecutive daily sessions. On the participant's first neuromodulation visit, rMT procedures were repeated in a treatment room. TMS was delivered at 110% of the AdjMT calculated using the scalp‐to‐cortex distance formula referred to above. Two rounds of TMS with a 5 min break in between were administered to the participant‐specific target using an MRI‐Image guided stereotaxic system (Rogue Research). Each round had 24 trains of 50 pulses for the pre‐determined frequency, totalling 2400 pulses for a single neuromodulation visit. Importantly, during TMS/fMRI sessions, stimulation was delivered at 120% of adjusted rMT to ensure robust and detectable BOLD responses within the limited duration of stimulation blocks, consistent with our prior work [51]. In contrast, neuromodulation sessions employed 110% of adjusted rMT to balance efficacy with participant comfort and tolerability during longer stimulation protocols.

Following rTMS, participants completed an RTI task and a DMTS task in a randomized order. This order was kept for all neuromodulation sessions. The tasks were practiced prior to any TMS during the first neuromodulation session. Pre and post‐TMS behavioral surveys were administered during each visit.

### 5.7 Brain Image Processing

The baseline structural MRI and functional MRI scans were pre‐processed using fMRIPrep 22.1.1 [52]. Structural images underwent intensity non‐uniformity correction with N4BiasFieldCorrection [53] from ANTs 2.3.3 [54], skull‐stripping with a Nipype implementation of the ANTs brain extraction workflow, and brain tissue segmentation with fast from FSL 6.0.5.1 [55]. Brain surfaces were then reconstructed using FreeSurfer 7.2.0 [56]. Volume‐based spatial normalization to two standard spaces (MNI152NLin2009cAsym, MNI152NLin6Asym) was performed through nonlinear registration with ANTs 2.3.3.

For the functional data, a skull‐stripped reference BOLD volume was generated through fMRIPrep. Head‐motion parameters with respect to the BOLD reference were calculated before any spatiotemporal filtering using mcflirt [57] from FSL. BOLD runs were slice‐time corrected using 3dTshift from AFNI [58, 59] and resampled onto their original, native space by applying the transform to correct for head‐motion. The BOLD reference was then co‐registered with rigid transformations to the T1‐weighted reference using bbregister [60] from FreeSurfer. The BOLD time‐series were resampled into standard space, generating a preprocessed BOLD run in MNI152NLin2009cAsym space. Automatic removal of motion artifacts using ICA‐AROMA [61] was performed on the preprocessed BOLD on the MNI space time‐series. All resamplings were performed with a single interpolation step by composing all the pertinent transformations. Post‐processing procedures by Nilearn [62], including detrend, low‐pass and high‐pass filtering, confounds removal (36 nuisance regressors), and spatial smoothing (6 mm FWHM), were applied to the processed functional data from fMRIPrep.

### 5.8 Personalized Functional Networks (PFNs)

PFNs are identified for each individual in this study to facilitate the brain readout computation and individualized target identification in the context of TMS and working memory. Specifically, a spatially regularized non‐negative matrix factorization technique was adopted for the identification of PFNs [27], which has been demonstrated to be capable of identifying FCNs in individuals accurately while maintaining inter‐individual correspondence. Since task fMRI data may provide information more sensitive to specific tasks than resting‐state fMRI data [63, 64, 65], we concatenated resting‐state and task fMRI in the temporal dimension to compute individualized FCNs. A consensus atlas was first constructed from concatenated resting‐state and N‐back task fMRI data from 90 independent healthy participants. PFNs were then derived based on the group atlas, following the procedure in prior work [66]. The number of PFNs was empirically set to 50, and one PFN was determined as a noise component by visual check and excluded from the analysis. The remaining forty‐nine group‐level PFNs are shown in Figure S1.

### 5.9 Real‐Time Brain State Decoder

To obtain fMRI‐based brain readout during the working memory task, we developed a deep learning (DL) based brain decoder. Particularly, we utilized the LSTM RNNs to learn mappings between fMRI data and brain states, which have previously demonstrated promising performance in fMRI‐based decoding tasks [35]. As schematically illustrated in Figure 2C, a predictive model of LSTM RNNs [31] was trained based on functional signatures extracted from PFNs to classify the brain activity as a 2‐back state or not during the working memory task fMRI session. The brain readout value was defined as the probability of being in a 2‐back state predicted by the decoder for the time points of interest.

Given a group of *n* subjects, each having a working memory task fMRI scan, and pre‐computed PFNs as described previously. Based on the PFNs, the functional signatures $F^{i}\in R^{T\times K}$ used for the brain decoding were defined as weighted mean time courses of the task fMRI data within PFNs. With the functional signatures of *n* subjects, an LSTM RNNs [31] model was built to predict the brain state of each time point based on its functional profile and the temporal dependency of its preceding time points. The architecture of the LSTM RNNs consisted of two hidden LSTM layers and one fully connected layer [35]. The hidden LSTM layers were used to encode the functional information with temporal dependency for each time point, and the fully connected layer was used to learn a mapping between the learned feature representation and the brain states. The functional signatures derived from PFNs were fed into the first LSTM layer as input features, and the hidden state vector output by the first LSTM layer was used as the input to the second LSTM layer. Each LSTM layer had 128 hidden nodes. A fully connected layer with 2 output nodes was adopted for predicting the brain states as 2‐back or not. Softmax cross‐entropy between real and predicted brain states was used to optimize the model weights during training.

N‐back working memory task‐fMRI data of 90 healthy participants from an independent dataset was utilized to train and evaluate the baseline decoder (90% for training and 10% for validation). Crucially, this independent dataset was acquired at the same institution and on the same MRI scanner as the primary study, and employed the same fractal‐based N‐back task paradigm described above, thereby minimizing inter‐site and inter‐paradigm variability and ensuring that differences in decoder performance could not be attributed to methodological inconsistencies across datasets. Specifically, we generated training samples by splitting the functional signatures of each training participant into clip matrices of 25 by 49 with an overlap of 20 time points between temporally consecutive training clips serving as data augmentation. We implemented the decoder using Tensorflow [67]. The ADAM optimizer with a learning rate of 0.001 was adopted, and the learning rate was updated every 20 000 training steps with a decay rate of 0.1. The total number of training steps was set to 100 000 with a batch size of 32. Once the decoder was trained, it could be applied to unseen fMRI data to obtain the brain readouts for certain time points of interest.

To alleviate the effects of the discrepancy between the image characteristics of TMS/fMRI and baseline fMRI data on the decoder, we further fine‐tune the decoder using TMS/fMRI data collected incrementally as the project proceeds, and the fine‐tuned decoder was applied to data of incoming unseen participants. Specifically, the pre‐computed individualized FCNs from baseline fMRI data for each participant were spatially registered to the native space of the TMS/fMRI data and used to extract the TMS/fMRI functional signatures. The functional signatures were then fed into the decoder to obtain the brain readouts at the time points of interest. Importantly, the decoder was fine‐tuned twice as the study progressed, yielding three decoder versions in total. In brief, the non‐fine‐tuned decoder (v1) was applied to the first 4 participants, as no study‐specific TMS/fMRI data were yet available. The decoder was then fine‐tuned using the collected TMS/fMRI data and applied to the subsequent 5 participants (v2). It was further fine‐tuned and applied to the remaining 10 participants (v3). To prevent data leakage, fine‐tuning was always performed exclusively on previously acquired data, never on data from participants to whom that decoder version was subsequently applied. Crucially, each participant received the same decoder version across all TMS/fMRI sessions (Visits 3, 7, and 11), ensuring that changes in brain readouts within a participant cannot be attributed to decoder differences. Outputs from the decoder were correlated with behavioral metrics, including accuracy, reaction time, and BIS, to understand what the decoder represents.

### 5.10 Individualized TMS Targeting

To identify an individualized TMS target that would facilitate working memory performance, we hypothesized that the potential TMS target was the brain region with the highest functional connectivity to working memory‐related PFNs. Accordingly, we first identified working memory‐related FCNs that contributed most to the performance of the working memory task decoding by analysing how the changes of functional signatures of PFNs affect the baseline brain decoder. We then derived the individualized targeting map based on FC analysis of these FCNs, and finally identified the targets based on the targeting map.

In this approach, given the trained baseline working memory decoder, a principal component analysis (PCA) based sensitivity analysis was carried out to identify the most significant PFNs for the working memory task decoding [35, 68]. Specifically, functional signatures of all PFNs were excluded one by one from the input, and the changes in decoding accuracy on testing participants were recorded, resulting in a change matrix encapsulating changes in working memory decoding performance. PCA was then applied to the change matrix to identify PCs that encoded the main directions of the prediction changes with respect to changes in the functional signatures of FCNs. The top five ranked sensitive PFNs, including frontoparietal networks and dorsal attentional networks, were considered to be most relevant to working memory tasks and subsequently used for target identification. Note that the PCA‐based sensitivity analysis used the baseline decoder trained on the independent 90‐participant dataset without fine‐tuning, as this decoder's feature weights reflect the general structure of working memory networks derived from a large, clean independent dataset. Fine‐tuning was applied only for real‐time decoding during TMS/fMRI sessions to account for participant‐specific signal characteristics and electromagnetic artefacts inherent to simultaneous TMS–fMRI acquisition.

With relevant PFNs identified, whole‐brain FC maps were computed for each PFN. For each voxel, FC was defined as the Pearson correlation coefficient between its time series and the PFN time course. An individualized FC map was then obtained for each participant by averaging the FC maps across all relevant PFNs, yielding a subject‐specific representation of FC strength. To improve robustness against noise in voxel‐wise FC estimates at the single‐subject level, candidate stimulation targets were constrained using a group‐level high‐FC mask. This mask was not used to define individual targets directly, but rather to restrict the spatial search space to regions consistently exhibiting high FC across participants. Specifically, the group‐level mask was derived by thresholding the group‐average FC map of all training participants at the 90th percentile. To ensure feasibility for the TMS application, a TMS‐accessibility mask was additionally applied. The final individualized targeting map was therefore defined as the intersection of (i) the participant‐specific FC map, (ii) the group‐level high‐FC mask, and (iii) the TMS accessibility mask. The individualized stimulation target was subsequently identified as the peak voxel within this constrained map, restricted to the gyral cortex. The gyrus region was determined by the cortical surface and sulcal depth measures generated by FreeSurfer [69]. While this approach improves reliability, it introduces a trade‐off between leveraging group‐level priors and fully capturing inter‐individual variability; future work should investigate fully individualised or adaptive targeting strategies to optimize this balance. Given the individualized target, E‐field modeling was utilized to determine the TMS coil orientation following the procedure in [70].

### 5.11 Statistical Analysis

All statistical analyses were performed in MATLAB R2023a (MathWorks) and IBM SPSS Statistics v29.0. Skipped Pearson correlations were computed with the Robust Correlation Toolbox.

Prior to analysis, variables were screened for completeness and distributional properties. Normality of paired‐difference scores was assessed using the Shapiro–Wilk test, and sphericity for repeated‐measures ANOVA was assessed with Mauchly's test, with Greenhouse–Geisser correction applied where violated. For correlations, influential observations were handled using the Skipped Pearson procedure, which combines iterative outlier detection with bootstrap inference; no observations were excluded from t‐tests or ANOVA models.

Continuous variables are reported as mean ± standard error (SE) in the main text and figures, unless otherwise stated. Error bars depict ± 1 SE; shaded bands on correlation plots depict 95% bootstrap confidence intervals (1000 resamples). Primary analyses were based on N = 19. Decoder version sub‐analyses used the participant subset assigned to each version (v1: n = 4; v2: n = 5; v3: n = 10), and the tCNR comparison used n = 10 (decoder‐classified) vs. n = 9 (behavioral fallback).

All tests were two‐tailed with α = 0.05. Paired‐samples t‐tests were used for within‐condition decoder comparisons. N‐back performance was analysed with one‐way repeated‐measures ANOVA; DMTS performance with three‐way repeated‐measures ANOVA (Stimulation Type × Day × Delay), decomposed via lower‐order ANOVAs and paired t‐tests where interactions were significant; RTI performance with two‐way repeated‐measures ANOVA (Stimulation Type × Day). Frequency‐specific effects on DMTS were assessed with a linear mixed‐effects model (Frequency, Day, Delay as fixed effects; Participant as random intercept). Effect sizes are reported as Cohen's d for pairwise contrasts, η^2^_p_ for ANOVA, Cohen's f^2^ for mixed‐effects fixed effects, and Skipped Pearson's r for correlations, each with exact p‐values and 95% confidence intervals. Post‐hoc pairwise comparisons were not alpha‐corrected because contrasts were specified a priori from the study's primary hypothesis; uncorrected p‐values and effect sizes are reported to allow transparent evaluation.

## Funding

This work was supported by the National Institutes of Health / National Institute of Mental Health (grant R01 MH120811 to D.J.O. and Y.F.); the Hart Fund in Cognitive Neuroscience (D.J.O.); the Penn‐CHOP Lifespan Brain Institute; the AE Foundation; and the Penn AI2D Center.

## Conflicts of Interest

The authors declare no conflict of interest.

## Supporting information




**Supporting File**: advs75975‐sup‐0001‐SuppMat.docx.

## Data Availability

Data supporting the findings of this study are available from the lead contact (D.J.O.) on reasonable request, subject to a data‐use agreement. The code for computing individualized functional networks is available at https://github.com/hmlicas/Collaborative_Brain_Decomposition and https://github.com/ZaixuCui/pncSingleFuncParcel/tree/master/Step_2nd_SingleParcellation. The analysis code for individualized TMS targeting and brain state decoder is available at https://github.com/hmlicas/Loop_TMS.
