# Supplementary material for: Personalized Network‐Guided Neuromodulation Enhances Human Working Memory
Source: Adv Sci (Weinh). 2026 Jun 12:e23009. Online ahead of print. doi: 10.1002/advs.202523009 (PMC13336854; doi:10.1002/advs.202523009)
Supplement: Supplementary file 1 — Supporting File: advs75975‐sup‐0001‐SuppMat.docx. [file ADVS-9999-e23009-s001.docx]

Supplementary Materials for

Personalized Network-Guided Neuromodulation Enhances Human Working Memory

Ahsan Khan*, Hongming Li, Camille Blaine, Julie Grier, Ethan Hammett, Almaris Figueroa-Gonzalez, Sarai Garcia, Romain Duprat, Justin Reber, Joseph Deluisi, Christos Davatzikos, Theodore D. Satterthwaite, Yong Fan, Desmond J. Oathes*

*Corresponding Authors Email: [ahsankhan@hkbu.edu.hk](mailto:ahsankhan@hkbu.edu.hk), [oathes@pennmedicine.upenn.edu](mailto:oathes@pennmedicine.upenn.edu)

**This file includes:**

Figures. S1 to S6

Tables S1 to S3

Supplementary Note S1


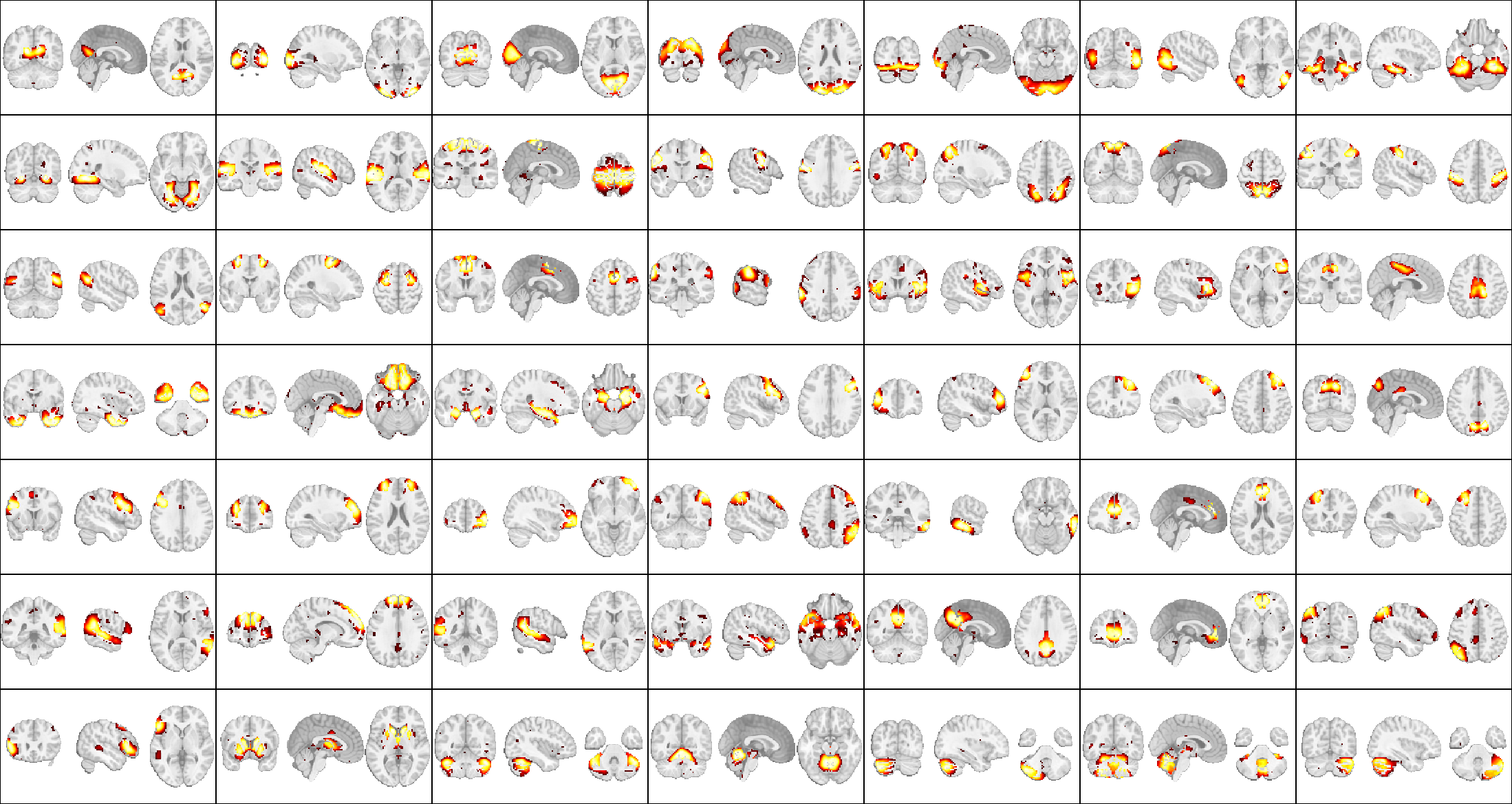


**Figure S1:** All FNs (group-level) identified by non-negative matrix factorization technique.


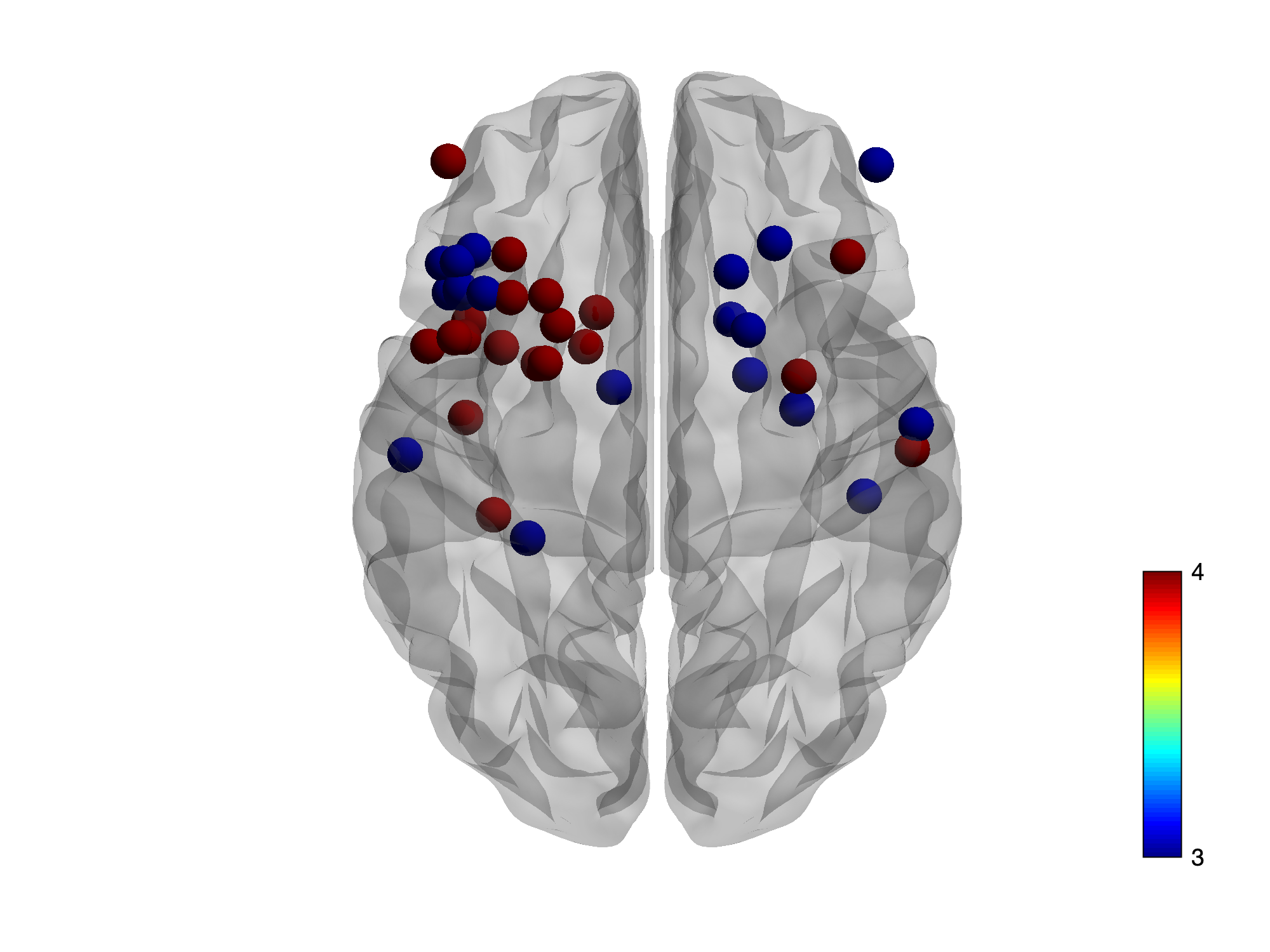


**Figure S2.** The figure illustrates the stimulation targets identified for each participant based on FCNs. Each participant has two targets: Target 1 serves as the primary target (highlighted in red), while Target 2 (highlighted in blue) acts as a backup in case the primary target is not reachable for stimulation.

**Supplementary Note S1: Decoder Failure Evaluation**

To explore potential patterns that lead to decoder failure, we evaluated the temporal contrast-to-noise ratio (tCNR) of the WM-relevant functional networks’ time courses from the pre-neuromodulation TMS/fMRI session. For each functional network, the tCNR of its time course was computed as $tCNR=\frac{abs(u_{2Back}-u_{0Back})}{(\sigma_{2Back}+\sigma_{0Back})/2}$, where $u_{2Back}$ and $u_{0Back}$ is the temporal mean of the 2-Back blocks and 0-Back blocks respectively, with $\sigma_{2Back}$ and $\sigma_{0Back}$ the temporal standard deviation. For each participant, the average tCNR across the WM-relevant functional networks was used for the comparison between the two decision-making groups (Decoder vs. Behavioral). Higher tCNR indicates better contrast between the 2-Back and 0-Back brain state.

The TMS/fMRI data of the participants in the Decoder group exhibited higher tCNR than that in the Behavioral group ($0.237\pm0.116$ vs. $0.172\pm0.058$) in general (Fig. R1), though the differences are not statistically significant ($p=0.075$, Two-sample *t*-test; $p=0.121$, Wilcoxon rank sum test). The relatively lower tCNR in the Behavioral group might limit the decoder performance to a certain extent.


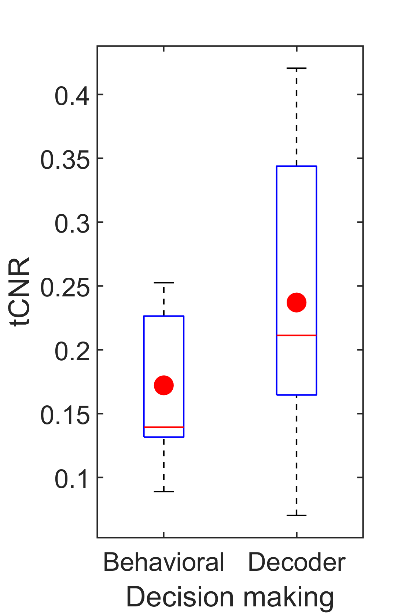


**Figure S3.** The temporal contrast-to-noise ratio (tCNR) of the WM-relevant functional networks’ time courses from the pre-neuromodulation TMS/fMRI session. The boxplot shows the distribution of tCNR across participants in two different decision-making groups, with “-” and “•” markers representing the median and mean, respectively.

**Figure S4.** The figure shows the behavioral results for each day during DMTS task including 0-sec, 4-sec, and 12-sec delays. No significant effects were observed for 0-delay condition, while for 4-sec and 12 sec condition participants were faster on Final Day of optimal stimulation.

**Figure S5: Reaction Time Across Stimulation Frequencies, Delay Conditions, and Visit Numbers**

Mean reaction times (± standard error) for correct responses across three experimental visits, stratified by stimulation frequency (5 Hz, 10 Hz, 20 Hz) and memory delay conditions (0, 4, 12 seconds). Each subplot corresponds to a distinct stimulation frequency, with visit number on the x-axis and separate bars representing delay conditions. Error bars reflect the standard error of the mean. The plots illustrate interaction effects between stimulation frequency, temporal delay, and visit number on task performance, derived from a linear mixed-effects model analysis.

**Figure S6: Accuracy Across Stimulation Frequencies, Delay Conditions, and Visit Numbers**
This grouped bar plot illustrates mean accuracy (± standard error) across three stimulation frequencies (5 Hz, 10 Hz, 20 Hz), three delay intervals (0s, 4s, 12s), and three neuromodulation sessions. Each subplot corresponds to a frequency condition, with bars grouped by delay and color-coded accordingly. Accuracy is expressed as a percentage, reflecting performance improvement across sessions and delay conditions. Error bars represent standard errors.

**Table S1**. provides the details of how the two runs of N-Back task were during Visit3.

Random Loop (random stimulation order)

no stimulation → 0-back, 2-back

first stim (**5**,10,20) → 2-back

second stim (5,**10**,20) → 2-back

third stim (5,10,**20**) → 2-back

no stimulation → 0-back, 2-back

fourth stim (5,10,**20**) → 2-back

fifth stim (5,**10**,20) → 2-back

sixth stim (**5**,10,20) → 2-back

**decides best, worst for the rest of the participant’s visits**

Informed Loop (closed loop stimulation order)

no stimulation → 0-back, 2-back

first stim (**best**) → 2-back

second stim (**worst**) → 2-back

third stim (**median**) → 2-back

no stimulation → 0-back, 2-back

fourth stim (**best of 1 v. 2. v 3**) → 2-back

fifth stim (**2nd best of 1 v. 2. v 3**) → 2-back

sixth stim (**best of 4 v. 5**) → 2-back

test frequency performance

**Table S2**. provides information about the frequencies identified as optimal and suboptimal for each of the participant who completed the study.

| Subjects | Optimal Frequency | Suboptimal Frequency | Visit 7 | Visit 11 | Decision Making |
| --- | --- | --- | --- | --- | --- |
| C106 | 10Hz | 20Hz | Optimal | Sub-optimal | Behavioral |
| C194 | 5Hz | 20Hz | Optimal | Sub-optimal | Behavioral |
| C236 | 20Hz | 10Hz | Sub-optimal | Optimal | Decoder |
| C397 | 5Hz | 10Hz | Sub-optimal | Optimal | Decoder |
| C435 | 5Hz | 20Hz | Sub-optimal | Optimal | Decoder |
| C475 | 10Hz | 20Hz | Optimal | Sub-optimal | Decoder |
| C527 | 20Hz | 10Hz | Optimal | Sub-optimal | Decoder |
| C537 | 10Hz | 5Hz | Optimal | Sub-optimal | Decoder |
| C542 | 5Hz | 10Hz | Sub-optimal | Optimal | Decoder |
| C549 | 20Hz | 10Hz | Sub-optimal | Optimal | Behavioral |
| C583 | 10Hz | 20Hz | Optimal | Sub-optimal | Decoder |
| C599 | 10Hz | 20Hz | Sub-optimal | Optimal | Behavioral |
| C605 | 10Hz | 20Hz | Sub-optimal | Optimal | Behavioral |
| C641 | 20Hz | 10Hz | Sub-optimal | Optimal | Behavioral |
| C694 | 10Hz | 5Hz | Sub-optimal | Optimal | Decoder |
| C775 | 5Hz | 20Hz | Optimal | Sub-optimal | Decoder |
| C844 | 5Hz | 10Hz | Sub-optimal | Optimal | Behavioral |
| C905 | 20Hz | 10Hz | Optimal | Sub-optimal | Behavioral |
| C976 | 5Hz | 10Hz | Optimal | Sub-optimal | Behavioral |

**Table S3**. provides the details of the run of N-Back task were during TMS/fMRI visit following optimal and suboptimal training.

Closed Loop (best, worst runs)

no stimulation → 0-back, 2-back

first stim (**optimal**) → 2-back

second stim (**suboptimal**) → 2-back

third stim (**optimal**) → 2-back

no stimulation → 0-back, 2-back

fourth stim (**suboptimal**) → 2-back

fifth stim (**optimal**) → 2-back

sixth stim (**suboptimal**) → 2-back
